# Quality circles for quality improvement in primary health care: Their origins, spread, effectiveness and lacunae– A scoping review

**DOI:** 10.1371/journal.pone.0202616

**Published:** 2018-12-17

**Authors:** Adrian Rohrbasser, Janet Harris, Sharon Mickan, Kali Tal, Geoff Wong

**Affiliations:** 1 Department of Continuing Education University of Oxford, Oxford, United Kingdom; 2 Institute of Primary Health Care (BIHAM), University of Bern, Bern, Switzerland; 3 University of Sheffield School of Health & Related Research, Sheffield, United Kingdom; 4 The Gold Coast Health, Griffith University, Southport, Australia; 5 Nuffield Department of Primary Care Health Sciences, University of Oxford, Oxford, United Kingdom; University of Antwerp, BELGIUM

## Abstract

Quality circles or peer review groups, and similar structured small groups of 6–12 health care professionals meet regularly across Europe to reflect on and improve their standard practice. There is debate over their effectiveness in primary health care, especially over their potential to change practitioners’ behaviour. Despite their popularity, we could not identify broad surveys of the literature on quality circles in a primary care context. Our scoping review was intended to identify possible definitions of quality circles, their origins, and reported effectiveness in primary health care, and to identify gaps in our knowledge. We searched appropriate databases and included any relevant paper on quality circles published until December 2017. We then compared information we found in the articles to that we found in books and on websites. Our search returned 7824 citations, from which we identified 82 background papers and 58 papers about quality circles. We found that they originated in manufacturing industry and that many countries adopted them for primary health care to continuously improve medical education, professional development, and quality of care. Quality circles are not standardized and their techniques are complex. We identified 19 papers that described individual studies, one paper that summarized 3 studies, and 1 systematic review that suggested that quality circles can effectively change behaviour, though effect sizes varied, depending on topic and context. Studies also suggested participation may affirm self-esteem and increase professional confidence. Because reports of the effect of quality circles on behaviour are variable, we recommend theory-driven research approaches to analyse and improve the effectiveness of this complex intervention.

## Introduction

Quality circles (QCs) or peer review groups, and other similar small groups of health care professionals meet regularly across Europe to reflect on and improve their standard practice. QCs are rooted in two fundamental concepts that shaped them from the beginning: the framework of the Plan-Do-Check-Act cycle, and the social context in which the group functions [1]. QCs use didactic methods like brain-storming and reflective thinking, and quality improvement (QI) techniques like audit and feedback or purposeful use of local experts. In several European countries, QCs support quality initiatives in primary health care (PHC) [2–11], as in Scotland and Wales, where structured small groups for QI were introduced to replace a pre-existing outcomes-driven incentive scheme [12, 13]. Many techniques QCs employ have been systematically reviewed but it is not clear if these techniques (alone or in combination) improve the practice of participants. This scoping review was intended to help us define QCs, describe their origin and intentions, explore their effectiveness in the context of PHC, and identify areas where there are gaps in our knowledge.

## Methods

### Method of the scoping search

Unlike systematic reviews, which are based on strictly defined research problems, scoping reviews usually address broadly formulated questions. They map literature on a broad topic to identify and describe studies, to look for definitions and identify and describe key concepts [14]. This approach relies on stepwise and iterative search techniques to develop a strategy to retrieve adequate literature. Inclusion and exclusion criteria and data extraction templates are not predetermined; instead, they develop gradually in tandem with the search [15, 16]. Scoping reviews do not formally assess the methodological quality of studies or data. They instead cast a broad net, capturing enough papers to begin to answer the broad questions they ask, laying the groundwork for later systematic reviews that can exclude papers of low methodological quality to improve validity [14, 17]. This broad focus allowed us to include and consult selected books and websites to supplement our literature search results [18].

The scoping search was conducted in several steps, following the guidelines for conducting systematic scoping reviews [19]:

- Identify literature on QCs and determine what kind of studies described and defined them;
- Determine the origin of QCs and how they spread;
- Describe their intentions and reported benefits;
- Review their reported effectiveness on behaviour change; and,
- Summarize questions unanswered in the literature.

### Information sources and search

The literature search was carried out by AR, who included all published articles up to December 2017. AR ran a limited search on the term ‘quality circle’ in PubMed to identify the first papers and then collaborated with an experienced librarian to expand the search. Together, they analysed text in the title and abstract and the article’s indexing terms to generate a broader list of terms. Iterative searching revealed descriptive terminology like ‘quality improvement’, ‘group functions’ and ‘primary care’ (S1 File). We retrieved literature from Medline, Embase, PsycInfo, and CINAHL without language or time restrictions and downloaded the citations to Endnote X8.

To check whether and how the definitions and processes described in the literature were implemented, AR searched websites in countries where the literature described active QCs [2–11]. If a website was not accessible to the public, AR contacted the authors or organisations to ask for access. AR, SM, and JH compared and discussed information from the literature and from the websites that described the definitions and processes of real-world QCs.

Because background information on underlying intentions, origin, and spread of QCs was scarce in published papers, AR looked for books published in countries where QC activities had been reported, searching in SOLO (Search Oxford Libraries Online: ‘quality circle’ and ‘peer review group’). Because QCs were introduced to PHC in the 1980s, we limited our search to books published after 1980, without language restrictions. We used filters including ‘education’, ‘knowledge management’ and ‘medical care’ to identify candidate books. Since few were available online, AR went to libraries to leaf through tables of contents, and, in consultation with SM and JH, selected those books that described the origin, definition, and processes of QCs. Books were included if they contained information on the origin and intentions of QC and if they described the basic characteristics of PHC QCs. We halted the search when we reached saturation and it was clear additional sources were no longer providing new information. We ultimately included 12 textbooks and used them to verify information retrieved from the literature identified in our database search (S2 File) [20–31].

### Eligibility criteria

We considered for inclusion any paper on QCs within PHC, in any language, with qualitative or quantitative outcomes, or background information. AR screened all papers identified by the search and SM, JH and GW cross-checked them to ensure eligibility criteria were consistently applied.

### Paper selection

We checked only to see if papers provided relevant information about QCs in PHC. AR assessed relevance and then discussed his findings with SM, JH and GW. Papers were relevant if they met criterium A or B:

A. The paper contained information about the background of QCs in PHC.

B. The paper described the process in these small groups and contained data to allow to evaluate QCs in PHC.

**Fig 1:**
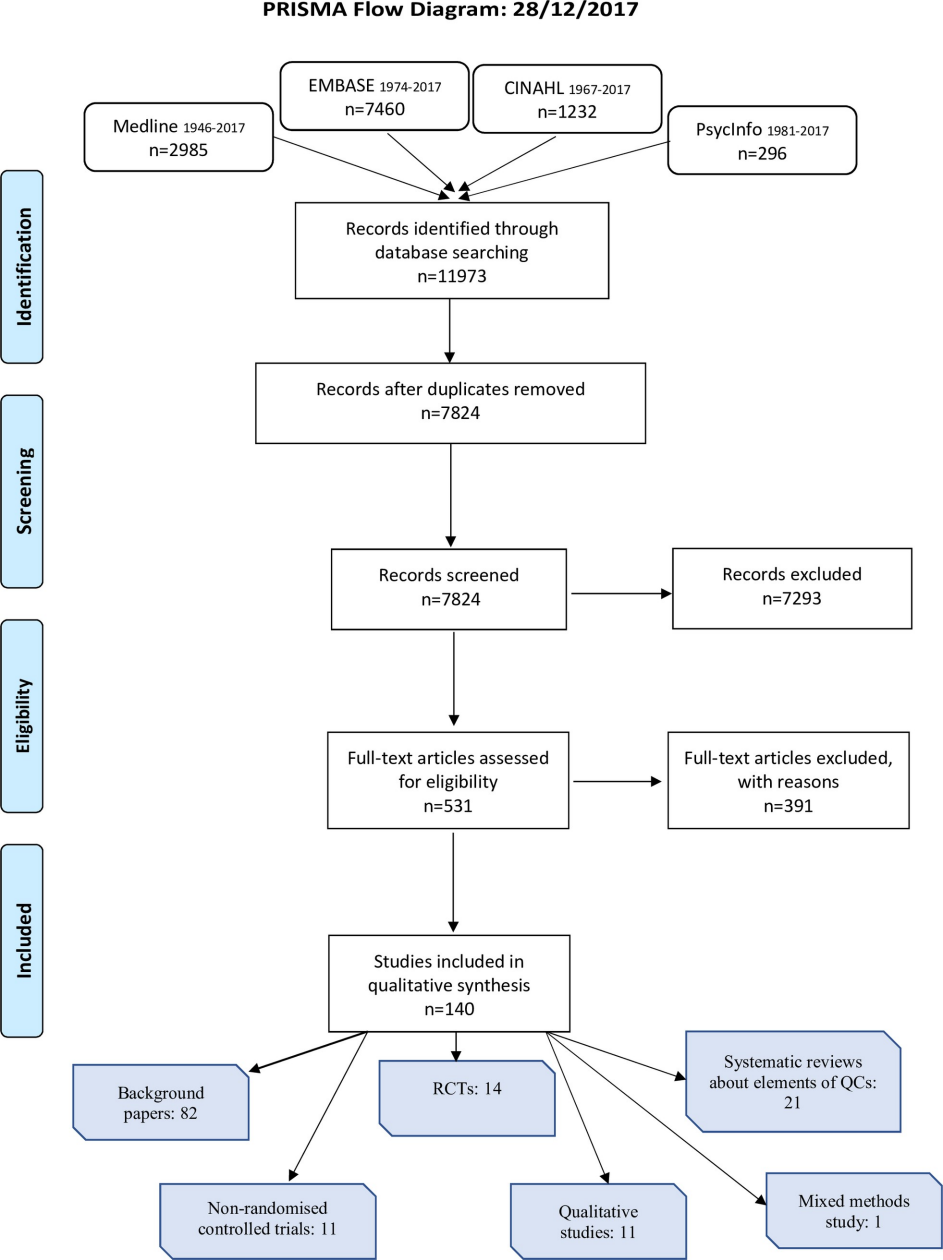
Paper flow diagram.

The flow diagram (Fig 1) shows the number of papers included and excluded at each stage.

### Data collection and reporting

We identified the aspects of the publications specific to study types and categorized them according to the Cochrane Manual [32]:

- randomized controlled trials, whether or not the nature of the intervention made blinding impossible
- non-randomised controlled trials, further grouped into controlled before-and-after studies, interrupted time series, historically controlled studies, cohort studies and case series (uncontrolled longitudinal studies)

AR extracted the following data: authors; publication year and location; descriptions of QC background; definitions of QCs; their underlying processes; their possible effectiveness; historical development; and, their spread. We used this data to generate a narrative and tables that describe the aspects of QCs. In parallel, we generated our data extraction template in Microsoft Excel 2016, taking an incremental approach. We then charted data for each topic, one at a time, to meet our objectives.

## Results

Our iterative searches returned 82 background papers (S3 File). Among retrieved papers, we deemed eligible and relevant 21 systematic reviews [33–53], 14 randomised controlled trials [54–67], 11 non-randomised controlled studies [68–78], 11 qualitative studies [79–89], and one mixed methods study [90] (S4 File). The systematic reviews, randomised controlled trials, cohort and controlled before-and-after studies each described and evaluated the processes or techniques QCs used. Qualitative studies and background papers described their processes and additional benefits. Background papers and the books and web resources we identified provided complementary information on the origin, definition, and spread of QCs.

### What quality circles are

We used the included papers to identify concurrent key concepts about QCs. All sources confirmed that QCs comprise small groups of 6–12 health care professionals who meet regularly to reflect on and improve their standard practice [2, 5–7, 9–11, 20, 22–29, 31, 71, 72, 78, 82, 91–97]. The terms Practice Based Small Group Work, Peer Review Group, Problem Based Small Group Learning, Practice Based Research Group, Quality Circle, Continuous Medical Education (CME) Group, and Continuous Professional Development (CPD) Group were used interchangeably and varied among countries. The labels suggest the basic, original intent of the group. We decided to use the umbrella term Quality Circle to describe all of them.

Terms like ‘peer review group’ or ‘*entre pairs*’ reflect the principle of equity in a group without a hierarchy. This group of equals creates a climate of trust that promotes a free speech culture where discussions of everyday problems are founded on collective expertise [10, 25, 95, 98]. It is similar to collegial counselling (intervision), where equals seek to solve an existing problem, e.g., when colleagues draw clinical cases and others help solve them. This is often the starting point for mutual learning [23, 96, 99]. Depending on the country’s tradition, QCs might not be limited to GPs but involve other professionals in PHC, including practice assistants (in the Netherlands and Germany) or practice teams (in Scotland), who add perspectives to the QC process [59, 67, 82, 100–102]. Interprofessional collaboration and mutual learning may also involve practice nurses [103] or specialists invited to QCs to share expertise on a specific topic, e.g., pharmacists who contribute to a discussion on prescription patterns [75, 104, 105].

Autonomy is another important principle [106]. The groups choose a topic they want to learn more about or an aspect of quality that they want to improve in their practice. They decide how to approach and solve the problem, and they create space to reflect on how to improve clinical practice [2, 6, 21, 28, 64, 78, 80, 84, 85, 98, 107–110]. The groups choose their own facilitators, who observe and lead the group through a QI cycle. QCs respect the contribution of each individual. They also consider group dynamics and try to keep members focused without controlling the discussion [25, 28, 43, 47, 79, 89, 111, 112].

QCs combine techniques, including discussing educational material in a workshop-like atmosphere, contact with local experts, auditing and feedback on clinical practice with or without outreach visits, facilitation, and local consensus processes [82, 84, 92, 93, 95, 97, 98, 113–118]. The group may also rehearse clinical skills and use active didactic methods to promote learning, including brain-storming, reflective thinking, self-monitoring and professionally reprocessing patient situations [2, 8, 9, 11, 24, 29, 31, 87].

Techniques and didactic methods are usually tailored to local contexts and circumstances. The number and difficulty of these techniques and didactic methods, and the outcomes and the context of the group, all affect the process [84, 87, 104]. QCs are therefore complex social interventions [119, 120] that run in PHC systems, constantly changing in response to new economic situations, scientific developments, and cultural pressures. They incorporate social aspects of the workplace that affect team work, self-determination and involvement in management at a day-to-day level.

### Origins and spread of quality circles

**Fig 2:**
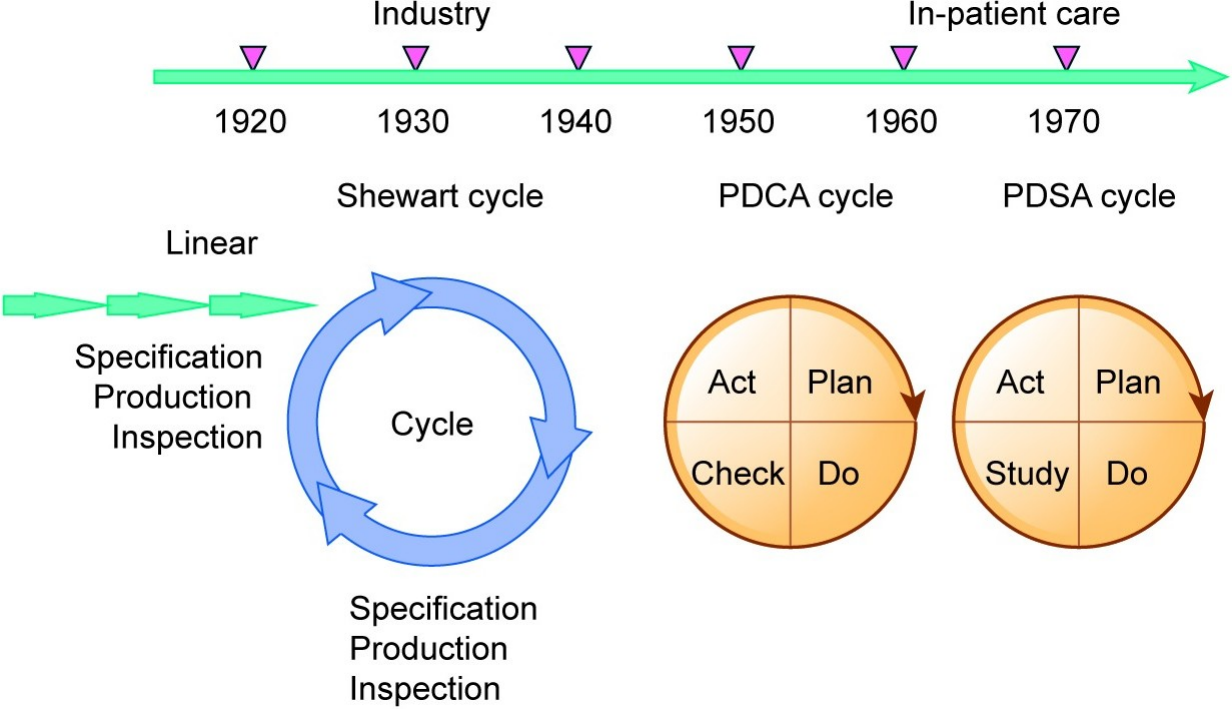
Development of the quality improvement process.

In 1924, Shewart created a table that depicted a cycle for continuous control of the QI process. Deming improved this model and introduced the Plan-Do-Check-Act cycle (Fig 2) [121]. The model was used by small groups of frontline workers instead of administrators because workers often know how to improve production. The assumption was that if workers volunteered to help develop the organization, they could improve both products and the work environment.

QCs spread first within manufacturing industry, then to the service industry, and finally to the medical sector. [20, 122]. Donabedian adopted the principles of QI to health care where there are also three interdependent quality dimensions: structure, process, and outcome [123]. His model of QI in health care was first implemented in in-patient settings and secondary-care clinics in the Netherlands. The development of QCs in health care was driven by a need for participative group problem-solving approaches and shared responsibility for decision-making in rapidly expanding and expensive health care systems [124]. QCs in PHC originated in two centres: McMaster University in Canada and the University of Nijmegen in the Netherlands. In their undergraduate programmes, both universities promoted Problem Based Learning (PBL), which confronts a group of learners with a problem they have to solve, so they must actively participate in learning about the related issues [125].

**Fig 3:**
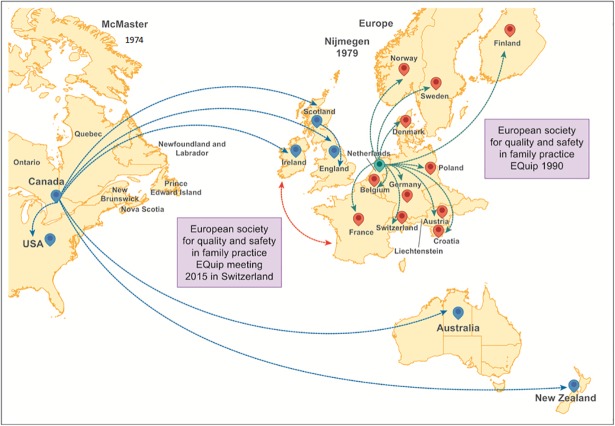
Spread of quality circles.

In 1974, at McMaster, Premi presented the results of 6 years’ experience of GPs who met on a regular basis to exchange thoughts about clinical cases and increase and update their knowledge [126]. This programme mainly addressed GPs’ needs for lifelong learning. As teachers, academics and policy makers built networks, the programme spread from McMaster, Canada, to Ireland, Scotland, and England and eventually to the USA, Australia and New Zealand as shown in Fig 3 [3, 7–9, 127].

In 1979, at the University of Nijmegen, Netherlands, PBL was implemented experimentally within small groups of GPs who met voluntarily on a regular basis to continuously and autonomously improve their knowledge through peer interaction [94]. Like Dutch hospitals had adopted Donabedian’s dimensions of quality in health care, the Dutch GPs adopted them in their small group work. Gradually, they transformed the learning cycle into a QI cycle, as their focus shifted from learning to improving practice [128, 129]. They combined didactic techniques from PBL with communication skills and understanding of group dynamics from industrial small group work. When the European Society for Quality and Safety in Family Medicine (EQuiP) was founded, it became a communication channel through which developments like QCs were shared. QCs then spread rapidly from the Netherlands to many other European countries (Fig 3) [2–11, 54, 69, 81, 83, 94, 130–135]. In 2015, EQuiP organised a conference in Fischingen, Switzerland, on QCs in PHC where representatives of these very similar movements documented the range of components they used in QCs, characterised their underlying mechanisms, and explored the local context in which they were conducted.

### Intentions and benefits of quality circles

Knowledge and skills acquired during early medical education must be regularly updated through continuous medical education, which helps medical professionals apply new knowledge via continuous professional development [33, 41, 136, 137]. CME and CPD are necessary prerequisites for QI [138–141]. QI is a data-guided activity that improves health care delivery by solving local problems like inefficient, harmful, or badly-timed health care [142, 143]. In some European countries, QCs seem to play a major role in QI; in others, they mainly serve CME and CPD [94].

The qualitative literature and background papers described the benefits of QCs. GPs seem to prefer learning in small groups [85, 103, 107, 140, 144] that help them to link evidence to everyday practice [79], learn to deal with uncertainty [81] and show them how to improve practice and feel secure in their professional roles [84]. QCs are a vehicle for discussing issues and reflecting on practice, which may raise self-esteem [83, 100]. Frequent participation strengthens team-based strategies for preventing errors [86]. When participants talk about their practice performance in groups, this can take them outside their comfort zone, causing anxiety and generating a stress response [83, 145]. But this stress response may improve communication skills and provide a learning opportunity [88, 89]. Several groups of authors note that working in small groups may help prevent burnout and give general practitioners a sense of belonging that so they changed workplace less often [51, 80, 89, 146–148].

### Reported effectiveness

We assessed 24 quantitative studies and one SR to determine if they claimed QCs promote behaviour change. Authors of four studies that examined guideline adherence reported their positive results had limited validity; four RCTs on the topic showed no effect, so the evidence on behaviour change concerning guideline adherence is not convincing. We found 15 papers, including one that summarized three studies and one SR, that suggest QCs may improve individual and group performance by reducing costs, encouraging professionals to order fewer but more appropriate tests, improving prescription habits, and reporting critical incidents. Reported effectiveness varied substantially within and among studies (Table 1).

**Table 1:** Effectiveness of quality circles.

First author/year	Study type	Intervention		Effective
		***Guideline adherence improved***		
Hartmann 1995 [68]	Controlled before-and-after	Diabetes type 2		(Yes)
Ioannidis 2007 [71]	Case series	Osteoporosis, pilot		(Yes)
Ioannidis 2009 [73]	Interrupted time series	Osteoporosis		Yes
Mahlknecht 2016 [78]	Case series	Chronic diseases		(Yes)
Elward 2014 [7])	controlled before-and-after	Asthma		(Yes)
Goldberg 1998 [54]	Randomised controlled	Hypertension and depression		No
Lagerlov 2000 [55]	Randomised controlled	Asthma and urinary tract infections		Yes
Schneider 2008 [60]	Randomised controlled	Asthma		No
Wilcock 2013 [64]	Randomised controlled	Dementia		No
Jager 2017 [67]	Randomised controlled	Polypharmacy		No
		***Prescription quality improved***		
Dyrkorn 2016 [77]	controlled before-and-after	for antibiotics		Yes
Welschen 2004 [59]	Randomised controlled	for antibiotics		Yes
Gjelstad 2013 [6]	Randomised controlled	for antibiotics		Yes
Vervloet 2016 [66]	Randomised controlled	for antibiotics		Yes
Rognstad 2013[63]	Randomised controlled	in general, for elderly		Yes
Richards 2003[69]	Historically controlled study	in general		Yes
		***Prescription quality improved and/or costs decreased***		
Wensing 2004 [70]	controlled before-and-after	prescription quality and costs		Yes
Wensing 2009 [7])	controlled before-and-after	prescription quality and costs		Yes
Niquille 2010 [75]	Cohort	prescription quality and costs		Yes
Riou 2007 [72]	Cohort	prescription costs		Yes
		***Test ordering quality improved and/or costs decreased***		
Verstappen 2003 [56]	Randomised controlled	test ordering quality		Yes
Verstappen 2004 [57]	Randomised controlled	test ordering quality		Yes
Verstappen 2004 [58]	Randomised controlled	test ordering quality and cost reduction		Yes
		***Patient safety improved***		
Verbakel 2015 [65]	Randomised controlled	reporting critical incidents		Yes
Zaher 2012 [51]	Systematic review	Behaviour change		Yes

() means that authors report limited validity of the results

SRs and one RCT show that facilitation enabled participants in QCs to introduce changes [43, 47] and that multifaceted interventions, peer review, audit, and feedback reinforce behaviour change [149] (Table 2).

**Table 2:** Systematic reviews and randomised controlled trials of techniques used in quality circles.

First author / year	Tool	Study type	Effect
**Predisposing**			
Davis 1999 [33]	Interactive CME meetings	SR	+
Davis 2006 [37]	Self-assessment	SR	-
O'Brien 2007 [38]	Educational outreach visits	SR	+
Bowie 2008 [39]	Significant event analysis	SR	+ / -
O’Brian 2001, Forsetlund 2009 [35, 41]	Educational meetings and workshops	SR	+
Harris 2011 [45]	Journal club	SR	+ / -
Flodgren 2011 [44]	Local opinion leaders	SR	+
Farmer 2008, Giguere 2012 [40, 48]	Printed educational materials	SR	+ / -
**Enabling**			
Grimshaw 2012 [49]	Clinical guidelines	SR	+ / -
Dogherty 2010, Baskerville 2012 [43, 47]	Facilitation	SR	++
Baker 2010, Baker 2015 [42, 52]	Tailored interventions	SR	+
Parmelli 2011 [46]	Change in organisational culture	SR	+/-
**Reinforcing**			
Gill 1999 [34]	Multifaceted interventions to improve prescribing	SR	+
Arnold 2005 [36]	Multifaceted interventions to improve antibiotic prescribing	SR	+
Roberts 2012 [61]	Peer review	RCT	+
Ivers 2012 [50]	Audit and feedback	SR	++
Cadogan 2015 [53]	Multifaceted interventions to improve test ordering	SR	+

+ /—no conclusive evidence

+ small effect

++ significant effect

### Summary of unanswered questions on quality circles addressed in the literature

Every author of an SRs that found QC techniques changed behaviour noted considerable variation within and between studies. They could document behaviour change in a SR, but not explain why it happened. SRs and RCTs that studied QC techniques only evaluated the impact or effectiveness of individual techniques but QCs often combine techniques and, in these cases, it is not clear how much each contributes to the overall effect [34, 36, 53].

To determine how and why techniques do or do not work, each step in the intervention process needs to be described in detail [38, 41, 44, 49] so we can evaluate the effectiveness of each step and each intervention, individually and in combination. For example, steps could include combining printed educational material with input from local opinion leaders, CME workshops, or outreach visits [48, 49].

We also need to account for the different contextual features of health care systems, and the roles these features play at each level. For instance, at the group level, professionals with different backgrounds may not all be equally involved in QI. At the institutional level, support for QC groups may vary. At the policy level, not all countries may leave QI to locally organised small groups [150]. We do not yet know which techniques should be used or what circumstances encourage QC participants to change their behaviour [52]. For example, audit and feedback interventions typically produce heterogeneous effects, and we would need to identify the underlying reasons for behaviour change after audit and feedback before we could know when to deliver this intervention, how best to design it, and how to optimise it in routine practice [50].

Small group work succeeds in continuous medical education, but we must ask how and why it could work or fail for quality projects [84]. What resources can small groups offer GPs to support changing their behaviour [73]? What it is about QCs that can improve the clinical performance of GPs? What group factors are crucial to better outcomes [74]. How frequently should group process should be repeated [50, 64, 65]? In their SR, Cadogan et al. argue that future research should be designed to improve our understanding of when, how, and why interventions like education or providing guidelines are likely to be effective and how these interventions can be improved. Such intervention studies should be based on a theory that can explain changes in clinical practice [53].

## Discussion

### Summary

QCs originated in industry and were implemented in health care after adapting aspects of quality critical for health care. QCs spread rapidly, since group work appears to meet GP expectations about CME, CPD and QI projects. As costs for health care have risen, so has the need for participatory, problem-solving group approaches and for shared responsibility. Reported benefits included giving participating professionals a better understanding of their roles, increasing their self-confidence and preventing burnout. But the reported effect of QCs on behaviour change varies substantially within and across studies, making interpretation of study results difficult.

### Limitations of retrieved papers

Most of the papers we reviewed found QCs had positive effects. This may suggest publication bias or outcome reporting bias [32]. Researcher allegiance could be one of the reasons for outcome reporting bias as it is likely that researchers and participants may have had a special interest in and were favourably disposed towards the QCs they examined. We found only one study that examined the performance of everyday activities of QCs [75], so data were mostly limited to interventions in newly formed groups. In existing QCs, researchers did not usually measure planned change, but performance after an intervention researcher introduced.

### Implications

Since QCs are a non-standardized complex intervention that varies by the topic and context of a group, inconsistent outcomes are unsurprising [151]. Complex interventions are hard to study, but realist approaches like realist review and realist evaluation could help us to make sense of QC outcomes [152–154]. These methods are designed to explain empirical outcomes and not just to quantify effect size. Since why and how QCs work is just as important as whether they can work, we need to understand the theoretical basis of interventions before we can explain why performance differs depending on the context, content, and application of QCs Theoretical models from other research fields like psychology and sociology could aid this exploration, since these also evolved analyse complex events and actions in different contexts. We have begun a realist review to fill some of these knowledge gaps [155].

### Strengths and limitations

Our review conforms to standard methods for scoping reviews and summarizes literature in all languages so it can guide future search and research strategies. Consulting varied sources allowed us to cross-check the information we gleaned from the articles. Because scoping reviews do not assess the methodological quality of included studies, our results are suggestive rather than conclusive.

## Conclusion

Quality circles originated in industry and migrated to health care where they meet the demands of general practitioners for continuous medical education, continuous professional development, and quality improvement. Quality circles may positively influence professional role perception and self-esteem, which could explain their broad, international adoption. But reported effects on prescribing behaviour or process changes vary substantially between studies, so we suggest a realist approach to exploring the constituents and contextual features of quality circles that improve performance

## Supporting information

S1 File Search strings. (DOCX)

S2 File Text books. (DOCX)

S3 File Background papers. (DOCX)

S4 File Papers examining quality circles. (DOCX)

## References

[1] Onglatco MU, Matsui T. The Anatomy of Japanese Quality Circles 1991 [Les circles de Qualité]. Available from: http://civilisations.revues.org/index1666.html.

[2] Diel F. Qualitätszirkel 2013 [German Definition of Structured Small Group Work]. Available from: http://www.kbv.de/html/qualitaetszirkel.php. German.

[3] Garbutt D, Dunion L, Walker S, Ford L, Steele D, Tannahill A, et al Welcome to the Practice Based Small Group Learning (PBSGL) Glasgow: National Health Service Scotland; 2015 [cited 2015 10/09]. PBSGL Home Page]. Available from: http://www.nes.scot.nhs.uk/education-and-training/by-theme-initiative/patient-safety-and-clinical-skills/safe-results/practice-based-small-group-learning-(pbsgl).aspx.

[4] Kirk UB. European Society of Quality and Safety in Primary Health Care (EQuiP): a network organisation within WONCA Region Europe 2015 [Available from: http://equip.woncaeurope.org/outputs/72-peer-reviewed-activities-2015-2011.

[5] Arvidsson GA, Elmroth U. PrimärvardsKvalitet—lanseringen fortsätter! [Quality in primary health care—the launch continues]. Allmän Medicin [Internet]. 2016 25/10/2016 [cited 2016 10/8]; 2 Available from: http://sfam.se/artiklar/primarvardskvalitet-lanseringen-fortsatter. Swedish.

[6] Hockl W. Qualitätszirkel 2016 [Austrian Definition of Structured Small Group Work]. Available from: https://oegam.at/qualitaetssicherung-und-qualitaetszirkel. German.

[7] Finnegan H. CME Small Group Meetings Dublin: Irish College of General Practitioners (ICPC); 2017 [Irish Structured Small Group Work]. Available from: https://www.icgp.ie/go/courses/cme_small_group_meetings.

[8] Elmslie T, Armson H, McLeod E, Bordma R, Shaw E, Teeple L, et al Practice Based Small Group Learning (PBSGL) Hamilton: McMaster; 2017 [Canadian Structured Small Group Work]. Available from: https://www.fmpe.org/.

[9] Rial J. Practice Based Small Group Learning (PBSGL) Southampton2017 [English Structured Small Group Work]. Available from: https://www.pbsgl.co.uk/.

[10] Dressarts T, Martin C. Les Groups de Pairs Paris: SGMF; 2017 [French Structured Small Group Work]. Available from: http://www.sfmg.org/groupe_de_pairs/. French.

[11] Rohrbasser A. Qualitätszirkel Bern: SSIM; 2017 [Swiss Definition of Structured Small Group Work]. Available from: http://www.sgaim.ch/de/qualitaet/qualitaetszirkel.html. German/French.

[12] Smith GI, Mercer SW, Gillies JC, McDevitt A. Improving together: a new quality framework for GP clusters in Scotland. British Journal of General Practice. 2017;67(660):294–5. 10.3399/bjgp17X691601 28663413 10.3399/bjgp17X691601 PMC5565863

[13] Rohrbasser A, Guthrie B, Gillies G, Mercer S. Collaborative Quality Improvement in General Practice Clusters. Report. Glasgow: Scottish School of Primary Care; 2017 03/08/2017. Contract No.: 12.

[14] Pham MT, Rajić A, Greig JD, Sargeant JM, Papadopoulos A, McEwen SA. A scoping review of scoping reviews: advancing the approach and enhancing the consistency. Research Synthesis Methods. 2014;5(4):371–85. 10.1002/jrsm.1123 26052958 10.1002/jrsm.1123 PMC4491356

[15] Levac D, Colquhoun H, O'Brien KK. Scoping studies: advancing the methodology. Implementation Science: IS. 2010;5:69–. 10.1186/1748-5908-5-69 20854677 10.1186/1748-5908-5-69 PMC2954944

[16] Moher D, Stewart L, Shekelle P. All in the Family: systematic reviews, rapid reviews, scoping reviews, realist reviews, and more. Systematic Reviews. 2015;4(1):183. 26693720 10.1186/s13643-015-0163-7 PMC4688988

[17] Armstrong R, Hall BJ, Doyle J, Waters E. ‘Scoping the scope’ of a cochrane review. Journal of Public Health. 2011;33(1):147–50. 10.1093/pubmed/fdr015 21345890 10.1093/pubmed/fdr015

[18] Arksey H, O'Malley L. Scoping studies: towards a methodological framework. International Journal of Social Research Methodology. 2005;8(1):19–32.

[19] Peters MDJ, Godfrey CM, Khalil H, McInerney P, Parker D, Soares CB. Guidance for conducting systematic scoping reviews. International Journal of Evidence-Based Healthcare. 2015;13(3):141–6. 10.1097/XEB.0000000000000050 26134548 10.1097/XEB.0000000000000050

[20] Ross JE, Ross WC. Japanese quality circles and productivity Reston, Va: Reston Pub. Co; 1982.

[21] Ishikawa K. How to Operate Quality Circle Activities. Tokyo: QC Headquarters, Union of Japanese Scientists and Engineers; 1985.

[22] Lawrence M, Schofield T. Medical Audit in Primary Health Care. Press OU, editor: Oxford University Press; 1993.

[23] Gerlach FM, Bahrs O. Qualitätssicherung durch hausärtzliche Qualitätszirkel: Strategien zur Etablierung. Bibliothek DD, editor. Berlin: Ullstein Mosby; 1994.

[24] Bahrs O, Gerlach FM, Szecsenyi J, editors. Ärztliche Qualitätszirkel: Leitfaden für den niedergelassenen Arzt. 2 ed Köln: Deutscher Ärzteverlag; 1995.

[25] Grol R, Lawrence M, editors. Quality Improvement by Peer Review. Oxford: Oxford University Press; 1995.

[26] Marinker M, editor. Medical Audit and General Practice Second edition ed. London: BMJ Publishing Group; 1995.

[27] Fraser R, Mayur L, Baker R. Evidence-Based Audit in General Practice Science E, editor: Butterworth Heinemann; 1999.

[28] Tross O. Qualitätszirkel als Form der Arbeitsorganisation: Planung und Gestaltung von Qualitätszirkeln als Variante der Teamarbeit in Unternehmen. GRIN, editor. München: Verlag für Akademische Texte; 2003.

[29] Elwyn G, Greenhalg T, Macfarlane F. Groups. A guide to small group work in healthcare, management, education and research: Radcliffe Medical Press; 2004.

[30] Saltman R, Bankauskaite V, Vrangbaek K. Primary care in the driver's seat?: Organizational reform in European primary care: McGraw-Hill Education (UK); 2005.

[31] Sommers LS, Launer J, editors. Clinical uncertainty in primary care: the challenge of collaborative engagement. London: Springer; 2013.

[32] Green S, Higgins J. Cochrane handbook for systematic reviews of interventions. Version; 2005.

[33] Davis D, O'Brien M, Freemantle N, Wolf FM, Mazmanian P, Taylor-Vaisey A. Impact of formal continuing medical education: Do conferences, workshops, rounds, and other traditional continuing education activities change physician behavior or health care outcomes? JAMA. 1999;282(9):867–74. 10478694 10.1001/jama.282.9.867

[34] Gill PS, Makela M, Vermeulen KM, Freemantle N, Ryan G, Bond C, et al Changing doctor prescribing behaviour. Pharmacy world & science: PWS. 1999;21(4):158–67. 10483603 10.1023/a:1008719129305

[35] O'Brien MA, Freemantle N, Oxman AD, Wolf F, Davis DA, Herrin J. Continuing education meetings and workshops: effects on professional practice and health care outcomes. Cochrane Database Syst Rev. 2001(2):CD003030 10.1002/14651858.CD003030 11406063 10.1002/14651858.CD003030

[36] Arnold SR, Straus SE. Interventions to improve antibiotic prescribing practices in ambulatory care. Cochrane Database Syst Rev. 2005(4):Cd003539 10.1002/14651858.CD003539.pub2 16235325 10.1002/14651858.CD003539.pub2 PMC7003679

[37] Davis DA, Mazmanian PE, Fordis M, Van Harrison R, Thorpe KE, Perrier L. Accuracy of physician self-assessment compared with observed measures of competence: a systematic review. JAMA. 2006;296(9):1094–102. 10.1001/jama.296.9.1094 16954489 10.1001/jama.296.9.1094

[38] O'Brien MA, Rogers S, Jamtvedt G, Oxman AD, Odgaard-Jensen J, Kristoffersen DT, et al Educational outreach visits: effects on professional practice and health care outcomes. Cochrane Database Syst Rev. 2007(4):Cd000409 10.1002/14651858.CD000409.pub2 17943742 10.1002/14651858.CD000409.pub2 PMC7032679

[39] Bowie P, Pope L, Lough M. A review of the current evidence base for significant event analysis. Journal of Evaluation in Clinical Practice. 2008;14(4):520–36. 10.1111/j.1365-2753.2007.00908.x 18462290 10.1111/j.1365-2753.2007.00908.x

[40] Farmer AP, Legare F, Turcot L, Grimshaw J, Harvey E, McGowan JL, et al Printed educational materials: effects on professional practice and health care outcomes. Cochrane Database Syst Rev. 2008(3):Cd004398 10.1002/14651858.CD004398.pub2 18646106 10.1002/14651858.CD004398.pub2

[41] Forsetlund L, Bjorndal A, Rashidian A, Jamtvedt G, O'Brien MA, Wolf F, et al Continuing education meetings and workshops: effects on professional practice and health care outcomes. Cochrane Database Syst Rev. 2009(2):Cd003030 10.1002/14651858.CD003030.pub2 19370580 10.1002/14651858.CD003030.pub2 PMC7138253

[42] Baker R, Camosso-Stefinovic J, Gillies C, Shaw EJ, Cheater F, Flottorp S, et al Tailored interventions to overcome identified barriers to change: effects on professional practice and health care outcomes. Cochrane Database Syst Rev. 2010(3):Cd005470 10.1002/14651858.CD005470.pub2 20238340 10.1002/14651858.CD005470.pub2 PMC4164371

[43] Dogherty EJ, Harrison MB, Graham ID. Facilitation as a Role and Process in Achieving Evidence-Based Practice in Nursing: A Focused Review of Concept and Meaning. Worldviews on Evidence-Based Nursing. 2010;7(2):76–89. 10.1111/j.1741-6787.2010.00186.x 20180826 10.1111/j.1741-6787.2010.00186.x

[44] Flodgren G, Parmelli E, Doumit G, Gattellari M, O'Brien MA, Grimshaw J, et al Local opinion leaders: effects on professional practice and health care outcomes. Cochrane Database Syst Rev. 2011(8):Cd000125 10.1002/14651858.CD000125.pub4 21833939 10.1002/14651858.CD000125.pub4 PMC4172331

[45] Harris J. KK, Henegan C. ME, N. R, R. P. Are journal clubs effective in supporting evidence-based decision making? A systematic review. BEME Guide No. 16. Medical Teacher. 2011;33(1):9–23. 10.3109/0142159X.2011.530321 21182379 10.3109/0142159X.2011.530321

[46] Parmelli E, Flodgren G, Schaafsma ME, Baillie N, Beyer FR, Eccles MP. The effectiveness of strategies to change organisational culture to improve healthcare performance. Cochrane Database Syst Rev. 2011(1):Cd008315 10.1002/14651858.CD008315.pub2 21249706 10.1002/14651858.CD008315.pub2 PMC4170901

[47] Baskerville NB, Liddy C, Hogg W. Systematic Review and Meta-Analysis of Practice Facilitation Within Primary Care Settings. The Annals of Family Medicine. 2012;10(1):63–74. 10.1370/afm.1312 22230833 10.1370/afm.1312 PMC3262473

[48] Giguere A, Legare F, Grimshaw J, Turcotte S, Fiander M, Grudniewicz A, et al Printed educational materials: effects on professional practice and healthcare outcomes. Cochrane Database Syst Rev. 2012;10:CD004398 10.1002/14651858.CD004398.pub3 23076904 10.1002/14651858.CD004398.pub3 PMC7197046

[49] Grimshaw JM, Schunemann HJ, Burgers J, Cruz AA, Heffner J, Metersky M, et al Disseminating and implementing guidelines: article 13 in Integrating and coordinating efforts in COPD guideline development. An official ATS/ERS workshop report. Proceedings of the American Thoracic Society. 2012;9(5):298–303. 10.1513/pats.201208-066ST 23256174 10.1513/pats.201208-066ST

[50] Ivers N, Jamtvedt G, Flottorp S, Young JM, Odgaard-Jensen J, French SD, et al Audit and feedback: effects on professional practice and healthcare outcomes. Cochrane Database Syst Rev. 2012(6):Cd000259 10.1002/14651858.CD000259.pub3 22696318 10.1002/14651858.CD000259.pub3 PMC11338587

[51] Zaher E, Ratnapalan S. Practice-based small group learning programs: systematic review. Can Fam Physician. 2012;58(6):637–42, e310-6. 22859626 PMC3374683

[52] Baker R, Camosso-Stefinovic J, Gillies C, Shaw EJ, Cheater F, Flottorp S, et al Tailored interventions to address determinants of practice. Cochrane Database Syst Rev. 2015(4):Cd005470 10.1002/14651858.CD005470.pub3 25923419 10.1002/14651858.CD005470.pub3 PMC7271646

[53] Cadogan SL, Browne JP, Bradley CP, Cahill MR. The effectiveness of interventions to improve laboratory requesting patterns among primary care physicians: a systematic review. Implement Sci. 2015;10:167 10.1186/s13012-015-0356-4 26637335 10.1186/s13012-015-0356-4 PMC4670500

[54] Goldberg HI, Wagner EH, Fihn SD, Martin DP, Horowitz CR, Christensen DB, et al A randomized controlled trial of CQI teams and academic detailing: can they alter compliance with guidelines? Joint Commission Journal on Quality Improvement. 1998;24(3):130–42. 9568553 10.1016/s1070-3241(16)30367-4

[55] Lagerlov P, Loeb M, Andrew M, Hjortdahl P. Improving doctors' prescribing behaviour through reflection on guidelines and prescription feedback: a randomised controlled study. Qual Health Care. 2000;9(3):159–65. 10.1136/qhc.9.3.159 10980076 10.1136/qhc.9.3.159 PMC1743532

[56] Verstappen WH, van der Weijden T, Sijbrandij J, Smeele I, Hermsen J, Grimshaw J, et al Effect of a practice-based strategy on test ordering performance of primary care physicians: a randomized trial. JAMA. 2003;289(18):2407–12. 10.1001/jama.289.18.2407 12746365 10.1001/jama.289.18.2407

[57] Verstappen WH, van der Weijden T, Dubois WI, Smeele I, Hermsen J, Tan FE, et al Improving test ordering in primary care: the added value of a small-group quality improvement strategy compared with classic feedback only. Ann Fam Med. 2004;2(6):569–75. 10.1370/afm.244 15576543 10.1370/afm.244 PMC1466745

[58] Verstappen WH, van Merode F, Grimshaw J, Dubois WI, Grol RP, van der Weijden T. Comparing cost effects of two quality strategies to improve test ordering in primary care: a randomized trial. Int J Qual Health Care. 2004;16(5):391–8. 10.1093/intqhc/mzh070 15375100 10.1093/intqhc/mzh070

[59] Welschen I, Kuyvenhoven MM, Hoes AW, Verheij TJ. Effectiveness of a multiple intervention to reduce antibiotic prescribing for respiratory tract symptoms in primary care: randomised controlled trial. BMJ. 2004;329(7463):431 10.1136/bmj.38182.591238.EB 15297305 10.1136/bmj.38182.591238.EB PMC514206

[60] Schneider A, Wensing M, Biessecker K, Quinzler R, Kaufmann-Kolle P, Szecsenyi J. Impact of quality circles for improvement of asthma care: results of a randomized controlled trial. J Eval Clin Pract. 2007;14(2):185–90. 10.1111/j.1365-2753.2007.00827.x 18093108 10.1111/j.1365-2753.2007.00827.x PMC2440309

[61] Roberts CM, Stone RA, Buckingham RJ, Pursey NA, Lowe D, Potter JM. A randomized trial of peer review: the UK National Chronic Obstructive Pulmonary Disease Resources and Outcomes Project: three-year evaluation. J Eval Clin Pract. 2012;18(3):599–605. 10.1111/j.1365-2753.2011.01639.x 21332611 10.1111/j.1365-2753.2011.01639.x

[62] Gjelstad S, Hoye S, Straand J, Brekke M, Dalen I, Lindbaek M. Improving antibiotic prescribing in acute respiratory tract infections: cluster randomised trial from Norwegian general practice (prescription peer academic detailing (Rx-PAD) study). BMJ. 2013;347:f4403 10.1136/bmj.f4403 23894178 10.1136/bmj.f4403 PMC3724398

[63] Rognstad S, Brekke M, Fetveit A, Dalen I, Straand J. Prescription peer academic detailing to reduce inappropriate prescribing for older patients: a cluster randomised controlled trial. Br J Gen Pract. 2013;63(613):e554–62. 10.3399/bjgp13X670688 23972196 10.3399/bjgp13X670688 PMC3722832

[64] Wilcock J, Iliffe S, Griffin M, Jain P, Thune-Boyle I, Lefford F, et al Tailored educational intervention for primary care to improve the management of dementia: The EVIDEM-ED cluster randomized controlled trial. Trials. 2013;14 (1) (no pagination)(397). 10.1186/1745-6215-14-397 PMC4222692 24257429

[65] Verbakel NJ, Langelaan M, Verheij TJM, Wagner C, Zwart DLM. Effects of patient safety culture interventions on incident reporting in general practice: A cluster randomised trial a cluster randomised trial. British Journal of General Practice. 2015;65(634):e319–e29. 10.3399/bjgp15X684853 25918337 10.3399/bjgp15X684853 PMC4408525

[66] Vervloet M, Meulepas MA, Cals JW, Eimers M, van der Hoek LS, van Dijk L. Reducing antibiotic prescriptions for respiratory tract infections in family practice: results of a cluster randomized controlled trial evaluating a multifaceted peer-group-based intervention. NPJ Primary Care Respiratory Medicine. 2016;26:15083 10.1038/npjpcrm.2015.83 26845640 10.1038/npjpcrm.2015.83 PMC4741286

[67] Jager C, Freund T, Steinhauser J, Stock C, Krisam J, Kaufmann-Kolle P, et al Impact of a tailored program on the implementation of evidence-based recommendations for multimorbid patients with polypharmacy in primary care practices-results of a cluster-randomized controlled trial. Implementation Science. 2017;12(1):8 10.1186/s13012-016-0535-y 28086976 10.1186/s13012-016-0535-y PMC5237147

[68] Hartmann P, Grusser M, Jorgens V. Strukturierte kassenarztliche Qualitatszirkel zum Thema Diabetikerbetreuung in der Praxis [Structured public health quality circle on the topic of diabetes management in general practice]. Zeitschrift fur arztliche Fortbildung. 1995;89(4):415–8. German. 7571745

[69] Richards D, Toop L, Graham P. Do clinical practice education groups result in sustained change in GP prescribing? Family Practice. 2003;20(2):199–206. 12651796 10.1093/fampra/20.2.199

[70] Wensing M, Broge B, Kaufmann-Kolle P, Andres E, Szecsenyi J. Quality circles to improve prescribing patterns in primary medical care: what is their actual impact? J Eval Clin Pract. 2004;10(3):457–66. 10.1111/j.1365-2753.2004.00517.x 15304146 10.1111/j.1365-2753.2004.00517.x

[71] Ioannidis G, Papaioannou A, Thabane L, Gafni A, Hodsman A, Kvern B, et al Canadian Quality Circle pilot project in osteoporosis: rationale, methods, and feasibility. Can Fam Physician. 2007;53(10):1694–700. 17934033 PMC2231434

[72] Riou F, Piette C, Durand G, Chaperon J. Results of a 12-month quality-circle prescribing improvement programme for GPs. Br J Gen Pract. 2007;57(540):574–6. 17727751 PMC2099641

[73] Ioannidis G, Papaioannou A, Thabane L, Gafni A, Hodsman A, Kvern B, et al The utilization of appropriate osteoporosis medications improves following a multifaceted educational intervention: the Canadian quality circle project (CQC). BMC Medical Education. 2009;9:54 10.1186/1472-6920-9-54 19660103 10.1186/1472-6920-9-54 PMC2731752

[74] Wensing M, Broge B, Riens B, Kaufmann-Kolle P, Akkermans R, Grol R, et al Quality circles to improve prescribing of primary care physicians. Three comparative studies. Pharmacoepidemiology and drug safety. 2009;18(9):763–9. 10.1002/pds.1778 19507170 10.1002/pds.1778

[75] Niquille A, Ruggli M, Buchmann M, Jordan D, Bugnon O. The nine-year sustained cost-containment impact of swiss pilot physicians-pharmacists quality circles. Ann Pharmacother. 2010;44(4):650–7. 10.1345/aph.1M537 20215496 10.1345/aph.1M537

[76] Elward K, Blackburn B, Peterson LE, Greenawald M, Hagen MD. Improving quality of care and guideline adherence for asthma through a group self-assessment module. Journal of the American Board of Family Medicine. 2014;27(3):391–8. 10.3122/jabfm.2014.03.130241 24808118 10.3122/jabfm.2014.03.130241

[77] Dyrkorn R, Gjelstad S, Espnes KA, Lindbaek M. Peer academic detailing on use of antibiotics in acute respiratory tract infections. A controlled study in an urban Norwegian out-of-hours service. Scandinavian Journal of Primary Health Care. 2016;34(2):180–5. 10.3109/02813432.2016.1163035 27054812 10.3109/02813432.2016.1163035 PMC4977941

[78] Mahlknecht A, Abuzahra ME, Piccoliori G, Enthaler N, Engl A, Sonnichsen A. Improving quality of care in general practices by self-audit, benchmarking and quality circles. Wiener klinische Wochenschrift. 2016;128(19–20):706–18. 10.1007/s00508-016-1064-z 27599700 10.1007/s00508-016-1064-z PMC5052301

[79] Watkins C, Timm A, Gooberman-Hill R, Harvey I, Haines A, Donovan J. Factors affecting feasibility and acceptability of a practice-based educational intervention to support evidence-based prescribing: a qualitative study. Fam Pract. 2004;21(6):661–9. 10.1093/fampra/cmh614 15528289 10.1093/fampra/cmh614

[80] Jenson CM, Hutchins AJ, Rowlands G. Is small-group education the key to retention of sessional GPs? Education for Primary Care. 2006;17(3):218–26. 10.1080/14739879.2006.11864065 28240104 10.1080/14739879.2006.11864065

[81] Sommers LS, Morgan L, Johnson L, Yatabe K. Practice inquiry: clinical uncertainty as a focus for small-group learning and practice improvement. J Gen Intern Med. 2007;22(2):246–52. 10.1007/s11606-006-0059-2 17356994 10.1007/s11606-006-0059-2 PMC1824750

[82] Overton GK, McCalister P, Kelly D, MacVicar R. The Practice-based Small Group Learning programme: experiences of learners in multi-professional groups. J Interprof Care. 2009;23(3):262–72. 10.1080/13561820802697628 19415563 10.1080/13561820802697628

[83] Frich J, Hoye S, Lindbaek M, Straand J. General practitioners and tutors' experiences with peer group academic detailing: a qualitative study. BMC Family Practice. 2010;11(1):12. 20152015 10.1186/1471-2296-11-12 PMC2828999

[84] Fisher DM, Brenner CJ, Cheren M, Stange KC. Engagement of groups in family medicine board maintenance of certification. Journal of the American Board of Family Medicine: JABFM. 2013;26(2):149–58. 10.3122/jabfm.2013.02.120262 23471928 10.3122/jabfm.2013.02.120262 PMC3814031

[85] Francois P, Philibert AC, Esturillo G, Sellier E. Groupes d’échange de pratique entre pairs: un modèle pour le développement professionnel continu en médecine générale [Peer groups: a model for the continuous professional development in general practice]. Presse Medicale. 2013;42(1):e21–7. French. 10.1016/j.lpm.2012.04.017 22721631

[86] Gehring K, Schwappach DLB, Battaglia M, Buff R, Huber F, Sauter P, et al Safety climate and its association with office type and team involvement in primary care. International Journal for Quality in Health Care. 2013;25(4):394–402. 10.1093/intqhc/mzt036 23667155 10.1093/intqhc/mzt036

[87] Andres E, Ludt S, Mainz A, Peters-Klimm F. 20 years of quality circles for family practitioners—Stocktaking and perspectives: A workshop report. [German]. Zeitschrift fur Allgemeinmedizin. 2015;91(2):66–70.

[88] Gehring SC, Kandzora J, Jeske-Saathoff E, Laag S, Hofmann W, Steinhauser J. Structured pharmacotherapy in multimorbid seniors—A pilot project. [German]. Zeitschrift fur Allgemeinmedizin. 2017;93(6):266–70.

[89] Nielsen HG, Davidsen AS. Witnesses in the consultation room—Experiences of peer group supervision. Education for Primary Care. 2017;28(5):258–64. 10.1080/14739879.2017.1300510 28317462 10.1080/14739879.2017.1300510

[90] Ter Brugge BPH, Bartelink MEL, Damoiseaux R, de Groot E. The use of evidence during group meetings of Dutch general practitioners. Education for Primary Care. 2017;28(6):307–12. 10.1080/14739879.2017.1344934 28701105 10.1080/14739879.2017.1344934

[91] Schillemans L, Grande LD, Remmen R. Using quality circles to evaluate the efficacy of primary health care. New Directions for Program Evaluation. 1989;1989(42):19–27.

[92] Gerlach FM, Beyer M, Romer A. Quality circles in ambulatory care: state of development and future perspective in Germany. Int J Qual Health Care. 1998;10(1):35–42. 10030785 10.1093/intqhc/10.1.35

[93] Ennis K, Harrington D. Quality management in Irish health care. Int J Health Care Qual Assur Inc Leadersh Health Serv. 1999;12(6–7):232–43. 10724566 10.1108/09526869910287305

[94] Beyer M, Gerlach FM, Flies U, Grol R, Krol Z, Munck A, et al The development of quality circles/peer review groups as a method of quality improvement in Europe. Results of a survey in 26 European countries. Fam Pract. 2003;20(4):443–51. 12876119 10.1093/fampra/cmg420

[95] Chop I, Eberlein-Gonska M. Übersichtsartikel zum Peer Review Verfahren und seine Einordnung in der Medizin [Overview on peer review techniques]. Zeitschrift für Evidenz, Fortbildung und Qualität im Gesundheitswesen. 2012;106(8):547–52. German. 10.1016/j.zefq.2012.08.017 23084858 10.1016/j.zefq.2012.08.017

[96] Armson H, Elmslie T, Roder S, Wakefield J. Encouraging Reflection and Change in Clinical Practice: Evolution of a Tool. Journal of Continuing Education in the Health Professions. 2015;35(3):220–31. 10.1002/chp.21299 26378428 10.1002/chp.21299

[97] Fuchs S, Parthier K, Wienke A, Mau W, Klement A. Fostering needs assessment and access to medical rehabilitation for patients with chronic disease and endangered work ability: Protocol of a multilevel evaluation on the effectiveness and efficacy of a CME intervention for general practitioners. Journal of Occupational Medicine and Toxicology. 2017;12 (1) (no pagination)(21). 10.1186/s12995-017-0168-3 PMC5545005 28785296

[98] Shears MR. Peer group learning in the context of an innovative postgraduate certificate for GP trainers: enhancing collaborative learning. Education for Primary Care. 2013;24(6):404–9. 24196596 10.1080/14739879.2013.11494210

[99] Eliasson G, Mattsson B. From teaching to learning. Experiences of small CME group work in general practice in Sweden. Scand J Prim Health Care. 1999;17(4):196–200. 10674295 10.1080/028134399750002403

[100] Overton GK, McCalister P, Kelly D, Macvicar R. Practice-based small group learning: how health professionals view their intention to change and the process of implementing change in practice. Med Teach. 2009;31(11):e514–20. 10.3109/01421590902842425 19909029 10.3109/01421590902842425

[101] Vollmar HC, Ostermann T, Hinz A, Rieger MA, Butzlaff ME. Hausarzte, Internet und Fortbildungsmedien. Nutzung und Effizienzeinschatzung durch Allgemeinarzte und hausarztlich tatige Internisten im 6-jahres-vergleich [Primary care physicians, internet and educational media. Preferences, usages and appraisal in a 6-year comparison] Medizinische Klinik. 2008;103(6):425–32. German. 10.1007/s00063-008-1055-6 18548212

[102] Jager C, Freund T, Steinhauser J, Joos S, Wensing M, Szecsenyi J. A tailored implementation intervention to implement recommendations addressing polypharmacy in multimorbid patients: Study protocol of a cluster randomized controlled trial. Trials. 2013;14 (1) (no pagination)(420). 10.1186/1745-6215-14-420 PMC4234123 24308282

[103] Overton GK, Kelly D, McCalister P, Jones J, MacVicar R. The practice-based small group learning approach: making evidence-based practice come alive for learners. Nurse Education Today. 2009;29(6):671–5. 10.1016/j.nedt.2009.02.009 19321240 10.1016/j.nedt.2009.02.009

[104] Davis MM, Keller S, DeVoe JE, Cohen DJ. Characteristics and lessons learned from practice-based research networks (PBRNs) in the United States. Journal of Healthcare Leadership. 2012;4:107–16. 10.2147/JHL.S16441 26213481 10.2147/JHL.S16441 PMC4512302

[105] Cunningham DE, Ferguson J, Wakeling J, Zlotos L, Power A. GP and pharmacist inter-professional learning—a grounded theory study. Education for Primary Care. 2016;27(3):188–95. 10.1080/14739879.2016.1163645 27022853 10.1080/14739879.2016.1163645

[106] Hömberg R, Voßschulte P. Qualitätszirkel: Selbstbestimmung geht verloren [Structured small group work: loss of autonomy]. Dtsch Arztebl International. 2010;107(36):1690–2. German.

[107] Lesmes-Anel J, Robinson G, Moody S. Learning preferences and learning styles: a study of Wessex general practice registrars. British Journal of General Practice. 2001;51(468):559–64. 11462316 PMC1314048

[108] Dahinden A, Rohrbasser A, Ryser O, Zoller M. Definition medizinischer Qualitätszirkel–ein Vernehmlassungstext Eine Neuorientierung der Empfehlungen für die medizinische Qualitätsarbeit in der Schweiz [Definition of structured small group work—acknowledged recommendations for quality improvement in Switzerland]. Primary Care. 2005;5(16):370–2. German.

[109] Jensen PM, Trollope-Kumar K, Waters H, Everson J. Building physician resilience. Can Fam Physician. 2008;54(5):722–9. 18474706 PMC2377221

[110] Zwald E. Die ARGOMED-Qualitätszirkel 2013 [Definition of Quality Circle in Networks]. Available from: http://www.argomed.ch/qualitaetszirkel.html.

[111] Weiss-Plumeyer m. Was sollte ein Moderator machen In: Bahrs O, Gerlach FM, Szecsenyi J, editors. Ärztliche Qualitätszirkel. 2 ed Köln: Deutscher Ärzte-Verlag; 1995 p. 97–108.

[112] MacVicar R, Guthrie V, O'Rourke J, Sneddon A. Supporting educational supervisor development at the interface: evaluation of a pilot of PBSGL for faculty development. Education for Primary Care. 2013;24(3):178–84. 23676873 10.1080/14739879.2013.11494169

[113] Davis DA, Thomson MA, Oxman AD, Haynes RB. Changing physician performance. A systematic review of the effect of continuing medical education strategies. JAMA. 1995;274(9):700–5. 7650822 10.1001/jama.274.9.700

[114] Oxman AD, Thomson MA, Davis DA, Haynes RB. No magic bullets: a systematic review of 102 trials of interventions to improve professional practice. CMAJ. 1995;153(10):1423–31. 7585368 PMC1487455

[115] Gerlach FM, Bahrs O, Weiss-Plumeyer M. [Quality circles in family practice—roots, concepts, perspectives]. Fortschr Med. 1994;112(8):56–61. 8194823

[116] Wakefield J, Herbert CP, Maclure M, Dormuth C, Wright JM, Legare J, et al Commitment to change statements can predict actual change in practice. J Contin Educ Health Prof. 2003;23(2):81–93. 10.1002/chp.1340230205 12866327 10.1002/chp.1340230205

[117] Anwar H, Batty H. Continuing Medical Education Strategy for Primary Health Care Physicians in Oman: Lessons to be learnt. Oman Medical Journal. 2007;22(3):33–5. 22400090 PMC3294157

[118] Niquille A, Ruggli M, Buchmann M, Jordan D, Bugnon O. The nine-year sustained cost-containment impact of Swiss pilot physicians-pharmacists quality circles. Annals of Pharmacotherapy. 2008;44 (4):650–7. 10.1345/aph.1M537 20215496

[119] Egan M, Bambra C, Thomas S, Petticrew M, Whitehead M, Thomson H. The psychosocial and health effects of workplace reorganisation. 1. A systematic review of organisational-level interventions that aim to increase employee control. Journal of Epidemiology and Community Health. 2007;61(11):945–54. 10.1136/jech.2006.054965 17933951 10.1136/jech.2006.054965 PMC2465601

[120] Mennin S. Small-group problem-based learning as a complex adaptive system. Teaching and Teacher Education. 2007;23(3):303–13.

[121] Best M, Neuhauser D. Walter A Shewhart, 1924, and the Hawthorne factory. Qual Saf Health Care. 2006;15(2):142–3. 10.1136/qshc.2006.018093 16585117 10.1136/qshc.2006.018093 PMC2464836

[122] Deming WE. Deming's 1950 Lecture to Japanese Management 1950 [Origin of PDSA Cycle]. Available from: http://hclectures.blogspot.com/1970/08/demings-1950-lecture-to-japanese.html.

[123] Donabedian A. 20 years of research on the quality of medical care, 1964–1984. Salud Publica Mex. 1988;30(2):202–15. 3137664

[124] Schmele JA, Allen ME, Butler S, Gresham D. Quality Circles in the Public Health Sector: Implementation and Effect. Public Health Nursing. 1991;8(3):190–5. 1946155 10.1111/j.1525-1446.1991.tb00754.x

[125] Wood DF. Problem based learning. BMJ. 2003;326(7384):328–30. 12574050 10.1136/bmj.326.7384.328 PMC1125189

[126] Premi JN. Continuing medical education in family medicine: a report of eight years' experience. Can Med Assoc J. 1974;111(11):1232–3. 4434295 PMC1955947

[127] Walsh AE, Armson H, Wakefield JG, Leadbetter W, Roder S. Using a novel small-group approach to enhance feedback skills for community-based teachers. Teaching and learning in medicine. 2009;21(1):45–51. 10.1080/10401330802574025 19130386 10.1080/10401330802574025

[128] Grol R, Baker R, Wensing M, Jacobs A. Quality Assurance in General Practice: the State of the Art in Europe. Family Practice. 1994;11(4):460–7. 7895977 10.1093/fampra/11.4.460

[129] Newton J, Hutchinson A, Steen N, Russell I, Haimes E. Educational potential of medical audit: observations from a study of small groups setting standards. Quality in health care: QHC. 1992;1(4):256–9. 10136875 10.1136/qshc.1.4.256 PMC1055037

[130] Thesen J. Kvalitetsverktoprosjektet 2010 [Quality improvement project 2010] Oslo: NFA; 2010 [updated 10/01/2010. Available from: http://legeforeningen.no/PageFiles/55104/120213%20Kvalitetsverkt%C3%B8yprosjektet.pdf. Norwegian.

[131] Goldberg HI, Rund DA, Hopkins JR. The Midpeninsula Health Service: action research using small primary care groups to provide evidence-based medicine that empowers patients while continuously improving quality and lowering costs. Medical care. 2002;40(4 Suppl):II32–9. 12064579

[132] Parker LE, de Pillis E, Altschuler A, Rubenstein LV, Meredith LS. Balancing participation and expertise: a comparison of locally and centrally managed health care quality improvement within primary care practices. Qualitative Health Research. 2007;17(9):1268–79. 10.1177/1049732307307447 17968043 10.1177/1049732307307447

[133] Williamson M, Cardona-Morrell M, Elliott JD, Reeve JF, Stocks NP, Emery J, et al Prescribing Data in General Practice Demonstration (PDGPD) project—a cluster randomised controlled trial of a quality improvement intervention to achieve better prescribing for chronic heart failure and hypertension. BMC Health Services Research. 2012;12:273 10.1186/1472-6963-12-273 22913571 10.1186/1472-6963-12-273 PMC3515472

[134] Griem C, Kleudgen S, Diel F. Qualitätssicherung: Instrumente der kollegialen Qualitätsförderung [Quality assurance: tool for collaborative quality improvement]. Dtsch Arztebl International. 2013;110(26):1310–3. German.

[135] McKnight A, Mills K. Continuing medical education for general practitioners—a Northern Ireland plan. Ulster Med J. 1992;61(2):157–62. 1481306 PMC2448934

[136] Nambiar RM. Professional development—in a changing world. Singapore medical journal. 2004;45(12):551–7. 15568115

[137] Davis D. Continuing education, guideline implementation, and the emerging transdisciplinary field of knowledge translation. J Contin Educ Health Prof. 2006;26(1):5–12. 10.1002/chp.46 16557510 10.1002/chp.46

[138] Czabanowska K, Klemenc-Ketis Z, Potter A, Rochfort A, Tomasik T, Csiszar J, et al Development of a competency framework for quality improvement in family medicine: a qualitative study. J Contin Educ Health Prof. 2012;32(3):174–80. 10.1002/chp.21142 23008079 10.1002/chp.21142

[139] Quasdorf I. Experience exchange in quality circles: No routine approach without recognized training [Erfahrungsaustausch in qualitatszirkeln: Kein Stammtisch, sondern anerkannte Fortbildung]. Deutsches Arzteblatt. 2008;105(5):A206–A9. German.

[140] Renschler HE. Methoden fur professionelles Weiterlernen: Ergebnis orientierender Umfragen bei Arzten. [Methods in continuing professional education: results of a pilot survey of physicians] Schweizerische Rundschau fur Medizin Praxis = Revue suisse de medecine Praxis. 1992;81(52):1574–85. German. 1475560

[141] Renschler HE. Systematic aspects of problem-based, case-related, practice-oriented, professional continuing education. [German] Systematik des problemorientierten, fallbezogenen, praxisgebundenen, professionellen Weiterlernens. Zeitschrift fur arztliche Fortbildung. 1995;89(4):392–6. 7571741

[142] Ogrinc G, Mooney SE, Estrada C, Foster T, Goldmann D, Hall LW, et al The SQUIRE (Standards for QUality Improvement Reporting Excellence) guidelines for quality improvement reporting: explanation and elaboration. Qual Saf Health Care. 2008;17(Suppl_1):i13–32. 18836062 10.1136/qshc.2008.029058 PMC2602740

[143] Glasziou P, Ogrinc G, Goodman S. Can evidence-based medicine and clinical quality improvement learn from each other? BMJ Qual Saf. 2011;20 Suppl 1:i13–7. 10.1136/bmjqs.2010.046524 PMC3066698 21450763

[144] Vollmar HC, Rieger MA, Butzlaff ME, Ostermann T. General Practitioners' preferences and use of educational media: a German perspective. BMC Health Serv Res. 2009;9:31 10.1186/1472-6963-9-31 19220905 10.1186/1472-6963-9-31 PMC2662827

[145] Henriksen K, Hansen EH. The threatened self: general practitioners' self-perception in relation to prescribing medicine. Soc Sci Med. 2004;59(1):47–55. 10.1016/j.socscimed.2003.10.004 15087142 10.1016/j.socscimed.2003.10.004

[146] Brondt A, Sokolowski I, Olesen F, Vedsted P. Continuing medical education and burnout among Danish GPs. British Journal of General Practice. 2008;58(546):15–9. 10.3399/bjgp08X263767 18186991 10.3399/bjgp08X263767 PMC2148233

[147] Kjaer NK, Steenstrup AP, Pedersen LB, Halling A. Continuous professional development for GPs: experience from Denmark. Postgraduate medical journal. 2014;90(1065):383–7. 10.1136/postgradmedj-2012-131679 24864203 10.1136/postgradmedj-2012-131679

[148] Peterson U, Bergstrom G, Samuelsson M, Asberg M, Nygren A. Reflecting peer-support groups in the prevention of stress and burnout: randomized controlled trial. Journal of advanced nursing. 2008;63(5):506–16. 10.1111/j.1365-2648.2008.04743.x 18727753 10.1111/j.1365-2648.2008.04743.x

[149] Woodward CA. Improving provider skills In: Organization WH, editor. Strategies for assisting health workers to modify and improve skills: Developing quality health care—a process of change. Geneva: Evidence and Information for Policy, Department of Organization of Health Services Delivery, World Health Organization; 2000.

[150] Rubenstein LV, Parker LE, Meredith LS, Altschuler A, DePillis E, Hernandez J, et al Understanding Team-based Quality Improvement for Depression in Primary Care. Health Services Research. 2002;37(4):1009–29. 10.1034/j.1600-0560.2002.63.x 12236381 10.1034/j.1600-0560.2002.63.x PMC1464007

[151] Walshe K. Understanding what works—and why—in quality improvement: the need for theory-driven evaluation. Int J Qual Health Care. 2007;19(2):57–9. 10.1093/intqhc/mzm004 17337518 10.1093/intqhc/mzm004

[152] Wong G. Getting to grips with context and complexity—the case for realist approaches. Gac Sanit. 2018;32(2):109–10. 10.1016/j.gaceta.2017.05.010 28735621 10.1016/j.gaceta.2017.05.010

[153] Wong G. Making theory from knowledge syntheses useful for public health. International Journal of Public Health. 2018. 10.1007/s00038-018-1098-2 29632957

[154] Keith RE, Crosson JC, O'Malley AS, Cromp D, Taylor EF. Using the Consolidated Framework for Implementation Research (CFIR) to produce actionable findings: a rapid-cycle evaluation approach to improving implementation. Implement Sci. 2017;12(1):15 10.1186/s13012-017-0550-7 28187747 10.1186/s13012-017-0550-7 PMC5303301

[155] Rohrbasser A, Mickan S, Harris J. Exploring why quality circles work in primary health care: a realist review protocol. Systematic Reviews. 2013;2(1):110. 24321626 10.1186/2046-4053-2-110 PMC4029275

