# Supplementary material for: Quality circles for quality improvement in primary health care: Their origins, spread, effectiveness and lacunae– A scoping review
Source: PLoS One. 2018 Dec 17;13(12):e0202616. doi: 10.1371/journal.pone.0202616 (PMC6296539; doi:10.1371/journal.pone.0202616)
Supplement: S1 File — (DOCX) [file pone.0202616.s001.docx]

**Search Strings: these three blocks are combined with the AND Boolean operator**

**“Quality Improvement” terms**

|  | Quality Improvement terms |  |
| --- | --- | --- |
| 1 | Quality Assurance, Health Care/Total Quality Management/ |  |
| 2 | Quality Improvement/ |  |
| 3 | "Quality of Health Care"/ |  |
| 4 | Evidence-based practice/ or evidence-based medicine/ or evidence-based nursing/ |  |
| 5 | Physician's Practice Patterns/ |  |
| 6 | exp Professional Competence/ |  |
| 7 | Guideline Adherence/ |  |
| 8 | (Quality adj3 (improv* or assurance or change)).ti,ab. |  |
| 9 | (Practice adj3 (improv* or change)).ti,ab. |  |
| 10 | ((Care or healthcare) adj3 (improv* or change)).ti,ab. |  |
| 11 | ((Professional or physician* or medical or clinical or nurs*) adj competenc*).ti,ab. |  |
| 12 | ((Guideline* or guidance or standard* or protocol*) adj2 (adhere* or complian* or concord* or implement*)).ti,ab. |  |
| 13 | (Evidence based adj2 (practice or prescrib*)).ti,ab. |  |
| 14 | 1 or 2 or 3 or 4 or 5 or 6 or 7 or 8 or 9 or 10 or 11 or 12 or 13 |  |
|  |  |  |

**“Group” terms**

|  | Group terms |
| --- | --- |
| 1 | Peer Groups/ |
| 2 | Group*.ti,ab |
| 3 | Group Process/ |
| 4 | Group Practice/ |
| 5  6  7  8 | Practice based.ti,ab  Facilitation.ti,ab  Facilitator.ti,ab  1 or 2 or 3 or 4 or 5 or 6 or 7 |

**“Primary Care” terms:**

|  | Primary Care Terms |  |
| --- | --- | --- |
| 1 | General practice/ or family practice/ |  |
| 2 | Primary Health Care/ |  |
| 3 | General practitioners/ or physicians, family/ or physicians, primary care/ |  |
| 4 | Community health services/ or community health nursing/ or community mental health services/ |  |
| 5 | (Family adj3 (practice or practitioner* or physician*)).ti,ab. |  |
| 6 | (General adj3 (practice or practitioner* or physician*)).ti,ab. |  |
| 7 | (Primary adj3 (care or healthcare)).ti,ab. |  |
| 8 | (Community adj2 nurs*).ti,ab. |  |
| 9 | 1 or 2 or 3 or 4 or 5 or 6 or 7 or 8 |  |
