# Supplementary material for: Quality circles for quality improvement in primary health care: Their origins, spread, effectiveness and lacunae– A scoping review
Source: PLoS One. 2018 Dec 17;13(12):e0202616. doi: 10.1371/journal.pone.0202616 (PMC6296539; doi:10.1371/journal.pone.0202616)
Supplement: S2 File — (DOCX) [file pone.0202616.s002.docx]

# Text books

Bahrs, O., et al., Eds. (1995). Ärztliche Qualitätszirkel: Leitfaden für den niedergelassenen Arzt. Köln, Deutscher Ärzteverlag.

Elwyn, G., et al. (2004). Groups. A guide to small group work in healthcare, management, education and research, Radcliffe Medical Press.

Fraser, R., et al. (1999). Evidence-Based Audit in General Practice Butterworth Heinemann.

Gerlach, F. M. and O. Bahrs (1994). Qualitätssicherung durch hausärtzliche Qualitätszirkel: Strategien zur Etablierung. Berlin, Ullstein Mosby.

Grol, R. and M. Lawrence, Eds. (1995). Quality Improvement by Peer Review. General Practices Series. Oxford, Oxford University Press.

Ishikawa, K. (1985). How to Operate Quality Circle Activities. Tokyo, QC Headquarters, Union of Japanese Scientists and Engineers.

Lawrence, M. and T. Schofield (1993). Medical Audit in Primary Health Care, Oxford University Press.

Marinker, M., Ed. (1995). Medical Audit and General Practice London, BMJ Publishing Group.

Ross, J. E. and W. C. Ross (1982). Japanese quality circles and productivity Reston, Va. , Reston Pub. Co.

Saltman, R., et al. (2005). Primary care in the driver's seat? Organizational reform in European primary care, McGraw-Hill Education (UK).

Sommers, L. S. and J. Launer, Eds. (2013). Clinical uncertainty in primary care: the challenge of collaborative engagement. London, Springer.

Tross, O. (2003). Qualitätszirkel als Form der Arbeitsorganisation: Planung und Gestaltung von Qualitätszirkeln als Variante der Teamarbeit in Unternehmen. München, Verlag für Akademische Texte.
