# Supplementary material for: Quality circles for quality improvement in primary health care: Their origins, spread, effectiveness and lacunae– A scoping review
Source: PLoS One. 2018 Dec 17;13(12):e0202616. doi: 10.1371/journal.pone.0202616 (PMC6296539; doi:10.1371/journal.pone.0202616)
Supplement: S3 File — (DOCX) [file pone.0202616.s003.docx]

# Background papers

**Anwar, H. and H.** Batty (2007). "Continuing Medical Education Strategy for Primary Health Care Physicians in Oman: Lessons to be learnt." Oman Medical Journal 22(3): 33-35.

**Armson, H., et al. (2015)**. "Encouraging Reflection and Change in Clinical Practice: Evolution of a Tool." Journal of Continuing Education in the Health Professions 35(3): 220-231.

**Armson, H., et al. (2015)**. "Is the Cognitive Complexity of Commitment-to-Change Statements Associated With Change in Clinical Practice? An Application of Bloom's Taxonomy." J Contin Educ Health Prof 35(3): 166-175.

**Armson, H. and J. Wakefield (2013**). "Expanding the horizons of practice-based small-group learning: what are we learning?" Education for Primary Care 24(3): 153-155.

**Best, M. and D. Neuhauser (2006)**. "Walter A Shewhart, 1924, and the Hawthorne factory." Qual Saf Health Care 15(2): 142-143.

**Beyer, M., et al. (2003).** "The development of quality circles/peer review groups as a method of quality improvement in Europe. Results of a survey in 26 European countries." Fam Pract 20(4): 443-451.

**Brennan, S., et al. (2012)**. "Measuring organizational and individual factors thought to influence the success of quality improvement in primary care: a systematic review of instruments." Implementation Science 7(1): 121.

**Brondt, A., et al. (2008)**. "Continuing medical education and burnout among Danish GPs." British Journal of General Practice 58(546): 15-19.

**Chop, I. and M. Eberlein-Gonska (2012)**. "Übersichtsartikel zum Peer Review Verfahren und seine Einordnung in der Medizin [Overview on peer review techniques]." Zeitschrift für Evidenz, Fortbildung und Qualität im Gesundheitswesen 106(8): 547-552.

**Cunningham, D. E., et al. (2016)**. "GP and pharmacist inter-professional learning - a grounded theory study." Education for Primary Care 27(3): 188-195.

**Czabanowska, K., et al. (2012)**. "Development of a competency framework for quality improvement in family medicine: a qualitative study." J Contin Educ Health Prof 32(3): 174-180.

**Dahinden, A., et al. (2005).** "Definition medizinischer Qualitätszirkel – ein Vernehmlassungstext Eine Neuorientierung der Empfehlungen für die medizinische Qualitätsarbeit in der Schweiz [Definition of structured small group work - acknowledged recommendations for quality improvement in Switzerland]." Primary Care 5(16): 370-372.

**Davis, D. (2000)**. "Clinical practice guidelines and the translation of knowledge: the science of continuing medical education." CMAJ 163(10): 1278-1279.

**Davis, D. (2006).** "Continuing education, guideline implementation, and the emerging transdisciplinary field of knowledge translation." J Contin Educ Health Prof 26(1): 5-12.

**Davis, M. M., et al. (2012).** "Characteristics and lessons learned from practice-based research networks (PBRNs) in the United States." Journal of Healthcare Leadership 4: 107-116.

**Donabedian, A. (1988).** "20 years of research on the quality of medical care, 1964-1984." Salud Publica Mex 30(2): 202-215.

**Egan, M., et al. (2007)**. "The psychosocial and health effects of workplace reorganisation. 1. A systematic review of organisational-level interventions that aim to increase employee control." Journal of Epidemiology and Community Health 61(11): 945-954.

**Eliasson, G. and B. Mattsson (1999)**. "From teaching to learning. Experiences of small CME group work in general practice in Sweden." Scand J Prim Health Care 17(4): 196-200.

**Ennis, K. and D. Harrington (1999).** "Quality management in Irish health care." Int J Health Care Qual Assur Inc Leadersh Health Serv 12(6-7): 232-243.

**Feron, J.-M., et al. (2003)**. "GPs working in solo practice: obstacles and motivations for working in a group? A qualitative study." Family Practice 20(2): 167-172.

**Fuchs, S., et al. (2017).** "Fostering needs assessment and access to medical rehabilitation for patients with chronic disease and endangered work ability: Protocol of a multilevel evaluation on the effectiveness and efficacy of a CME intervention for general practitioners." Journal of Occupational Medicine and Toxicology 12 (1) (no pagination)(21).

**Gerlach, F. M., et al. (1998).** "Quality circles in ambulatory care: state of development and future perspective in Germany." Int J Qual Health Care 10(1): 35-42.

**Glasziou, P., et al. (2011).** "Can evidence-based medicine and clinical quality improvement learn from each other?" BMJ Qual Saf 20 Suppl 1: i13-17.

**Goulet, F., et al. (2013)**. "Effects of continuing professional development on clinical performance: results of a study involving family practitioners in Quebec." Canadian Family Physician 59(5): 518-525.

**Griem, C., et al. (2013**). "Qualitätssicherung: Instrumente der kollegialen Qualitätsförderung [Quality assurance: tool for collaborative quality improvement]." Dtsch Arztebl International 110(26): 1310-1313.

**Grol, R., et al. (1994)**. "Quality Assurance in General Practice: the State of the Art in Europe." Family Practice 11(4): 460-467.

**Hanel, P., et al. (2014)**. "SIQ: Didactic support for quality circles in Saxony-Anhalt. [German]." Zeitschrift fur Allgemeinmedizin 90(2): 66-71.

**Henriksen, K. and E. H. Hansen (2004)**. "The threatened self: general practitioners' self-perception in relation to prescribing medicine." Soc Sci Med 59(1): 47-55.

**Hömberg, R. and P. Voßschulte (2010)**. "Qualitätszirkel: Selbstbestimmung geht verloren [Structured small group work: loss of autonomy]." Dtsch Arztebl International 107(36): 1690-1692.

**Ishikawa, K. (1985)**. How to Operate Quality Circle Activities. Tokyo, QC Headquarters, Union of Japanese Scientists and Engineers.

**Jager, C., et al. (2013)**. "A tailored implementation intervention to implement recommendations addressing polypharmacy in multimorbid patients: Study protocol of a cluster randomized controlled trial." Trials 14 (1) (no pagination)(420).

**Jansen, P. and I. Schluckebier (2007)**. "Quality circles for physician assistants: Implementation and continuous evaluation. [German] Qualitatszirkel fur arzthelferinnen: Implementierung und kontinuierliche evaluation." Zeitschrift fur Allgemeinmedizin 83(4): 143-147.

**Jensen, P. M., et al. (2008)**. "Building physician resilience." Can Fam Physician 54(5): 722-729.

**Joos, S., et al. (2005)**. "ELSID-Diabetes study-evaluation of a large scale implementation of disease management programmes for patients with type 2 diabetes. Rationale, design and conduct - A study protocol [ISRCTN08471887]." BMC Public Health 5(99).

**Kember, D. (1999).** "Determining the level of reflective thinking from students' written journals using a coding scheme based on the work of Mezirow." International Journal of Lifelong Education 18(1): 18-30.

**Kinn, S. (1997).** "The relationship between clinical audit and ethics." Journal of Medical Ethics 23(4): 250-253.

**Kjaer, N. K., et al. (2014).** "Continuous professional development for GPs: experience from Denmark." Postgrad Med J 90(1065): 383-387.

**Korzilius, H. (2013).** "Symposium zur Qualitätssicherung: Der Erfolg ist messbar [Symposium on quality assurance: success is measurable]." Dtsch Arztebl International 110(19): 920.

**Kotecha, J., et al. (2015).** "The role of the practice facilitators in Ontario primary healthcare quality improvement." BMC Family Practice 16: 93.

**Lesmes-Anel, J., et al. (2001)**. "Learning preferences and learning styles: a study of Wessex general practice registrars." British Journal of General Practice 51(468): 559-564.

**Lockyer, J., et al. (2011).** "Feedback data sources that inform physician self-assessment." Med Teach 33(2): e113-120.

**MacVicar, R., et al. (2013)**. "Supporting educational supervisor development at the interface: evaluation of a pilot of PBSGL for faculty development." Education for Primary Care 24(3): 178-184.

**Matsui, T. and M. U. Onglatco (1990)**. "Relationships between employee quality circle involvement and need fulfillment in work as moderated by work type: A compensatory or a spillover model?" Kleinbeck, Uwe [Ed]: 191-199.

**McKnight, A. and K. Mills (1992).** "Continuing medical education for general practitioners--a Northern Ireland plan." Ulster Med J 61(2): 157-162.

**Mennin, S. (2007)**. “Small-group problem-based learning as a complex adaptive system”

Teaching and Teacher Education 23(3): 303-313

**Nambiar, R. M. (2004).** "Professional development--in a changing world." Singapore Med J 45(12): 551-557.

**Newton, J., et al. (1992).** "Educational potential of medical audit: observations from a study of small groups setting standards." Quality in health care : QHC 1(4): 256-259.

**Ogrinc, G., et al. (2016)**. "SQUIRE 2.0 (Standards for QUality Improvement Reporting Excellence): revised publication guidelines from a detailed consensus process." BMJ Qual Saf 25(12): 986-992.

**Ogrinc, G., et al. (2008).** "The SQUIRE (Standards for QUality Improvement Reporting Excellence) guidelines for quality improvement reporting: explanation and elaboration." Qual Saf Health Care 17(Suppl_1): i13-32.

**Onglatco, M. U. and T. Matsui (1991)**. "The Anatomy of Japanese Quality Circles." Civilisations. Retrieved 04.12.2011, 2011, from http://civilisations.revues.org/index1666.html.

**Overton, G. K., et al. (2009)**. "The practice-based small group learning approach: making evidence-based practice come alive for learners." Nurse Education Today 29(6): 671-675.

**Overton, G. K., et al. (2009).** "Practice-based small group learning: how health professionals view their intention to change and the process of implementing change in practice." Med Teach 31(11): e514-520.

**Ovretveit, J. and D. Gustafson (2003).** "Using research to inform quality programmes." BMJ 326(7392): 759-761.

**Oxman, A. D., et al. (1995).** "No magic bullets: a systematic review of 102 trials of interventions to improve professional practice." CMAJ 153(10): 1423-1431.

**Pagliari, C. and J. Grimshaw (2002)**. "Impact of group structure and process on multidisciplinary evidence-based guideline development: an observational study." J Eval Clin Pract 8(2): 145-153.

**Parboosingh, J. T. (2002).** "Physician communities of practice: where learning and practice are inseparable." Journal of Continuing Education in the Health Professions 22(4): 230-236.

**Parker, L. E., et al. (2007)**. "Balancing participation and expertise: a comparison of locally and centrally managed health care quality improvement within primary care practices." Qualitative Health Research 17(9): 1268-1279.

**Payne, V. L. and S. J. Hysong (2016)**. "Model depicting aspects of audit and feedback that impact physicians' acceptance of clinical performance feedback." BMC Health Serv Res 16: 260.

**Pereles, L., et al. (2002).** "Permanent small groups: group dynamics, learning, and change." J Contin Educ Health Prof 22(4): 205-213.

**Peterson, U., et al. (2008).** "Reflecting peer-support groups in the prevention of stress and burnout: randomized controlled trial." J Adv Nurs 63(5): 506-516.

**Quasdorf, I. (2008).** "Experience exchange in quality circles: No routine approach without recognized training. [German] Erfahrungsaustausch in qualitatszirkeln: Kein stammtisch, sondern anerkannte fortbildung." Deutsches Arzteblatt 105(5): A206-A209.

**Renschler, H. E. (1992).** "Methods in continuing professional education. Results of a pilot survey of physicians. [German] Methoden fur professionelles Weiterlernen. Ergebnis orientierender Umfragen bei Arzten." Schweizerische Rundschau fur Medizin Praxis = Revue suisse de medecine Praxis 81(52): 1574-1585.

**Rohrbasser, A., et al. (2017)**. Collaborative Quality Improvement in General Practice Clusters. Briefing Papers. S. S. o. P. Care. Glasgow, Scottish School of Primary Care. 12: 7.

**Rubenstein, L. V., et al. (2002)**. "Understanding Team-based Quality Improvement for Depression in Primary Care." Health Services Research 37(4): 1009-1029.

**Sampson, R., et al. (2017).** "Improving the primary-secondary care interface in Scotland: a qualitative exploration of impact on clinicians of an educational complex intervention." BMJ Open 7(6).

**Schillemans, L., et al. (1989).** "Using quality circles to evaluate the efficacy of primary health care." New Directions for Program Evaluation 1989(42): 19-27.

**Schmele, J. A., et al. (1991).** "Quality Circles in the Public Health Sector: Implementation and Effect." Public Health Nursing 8(3): 190-195.

**Schubert, I., et al. (2009).** "Gelesen ist noch nicht getan: Hinweise zur Akzeptanz von hausärztlichen Leitlinien. Eine Befragung in Zirkeln der Hausarztzentrierten Versorgung (HZV)[Read is not implemented: information on acceptance of guidelines in primary care . A survey]." Zeitschrift für Evidenz, Fortbildung und Qualität im Gesundheitswesen 103(1): 5-12.

**Schubert, I., et al. (2010).** "General practitioners' guideline for palliative care. A survey of guideline acceptance in quality circles of primary medical care. [German] Hausarztliche Leitlinie "Palliativversorgung" : Ergebnisse einer Akzeptanzbefragung in Qualitatszirkeln der Hausarztzentrierten Versorgung." Medizinische Klinik (Munich, Germany : 1983) 105(3): 135-141.

**Scott, A., et al. (2011)** The effect of financial incentives on the quality of health care provided by primary care physicians. Cochrane Database of Systematic Reviews DOI: 10.1002/14651858.CD008451.pub2

**Shears, M. R. (2013).** "Peer group learning in the context of an innovative postgraduate certificate for GP trainers: enhancing collaborative learning." Education for Primary Care 24(6): 404-409.

**Smith, G. I., et al. (2017).** "Improving together: a new quality framework for GP clusters in Scotland." British Journal of General Practice 67(660): 294-295.

**Solomons, N. M. and J. A. Spross (2010).** "Evidence-based practice barriers and facilitators from a continuous quality improvement perspective: an integrative review." Journal of Nursing Management 19(1): 109-120.

**Stevenson, K., et al. (2001).** "Features of primary health care teams associated with successful quality improvement of diabetes care: a qualitative study." Fam Pract 18(1): 21-26.

**van den Hombergh, P., et al. (1999).** "Practice visits as a tool in quality improvement: acceptance and feasibility." Qual Health Care 8(3): 167-171.

**Vollmar, H. C., et al. (2008)**. "Primary care physicians, internet and educational media. Preferences, usages and appraisal in a 6-year comparison. [German] Hausarzte, internet und fortbildungsmedien. Nutzung und effizienzeinschatzung durch allgemeinarzte und hausarztlich tatige internisten im 6-jahresvergleich." Medizinische Klinik 103(6): 425-432.

**Vollmar, H. C., et al. (2009).** "General Practitioners' preferences and use of educational media: a German perspective." BMC Health Serv Res 9: 31.

**Walsh, A. E., et al. (2009).** "Using a novel small-group approach to enhance feedback skills for community-based teachers." Teach Learn Med 21(1): 45-51.

**Williamson, M., et al. (2012).** "Prescribing Data in General Practice Demonstration (PDGPD) project--a cluster randomised controlled trial of a quality improvement intervention to achieve better prescribing for chronic heart failure and hypertension." BMC Health Services Research 12: 273.

**Wood, D. F. (2003).** "Problem based learning." BMJ 326(7384): 328-330.

**Woodward, C. A. (2000).** Improving provider skills. Strategies for assisting health workers to modify and improve skills: Developing quality health care - a process of change. W. H. Organization. Geneva, Evidence and Information for Policy, Department of Organization of Health Services Delivery, World Health Organization. 1.

**Wynia, M. K. 1999.** Performance measures for ethics quality. *Eff Clin Pract,* 2**,** 294-8.
