# Supplementary material for: Quality circles for quality improvement in primary health care: Their origins, spread, effectiveness and lacunae– A scoping review
Source: PLoS One. 2018 Dec 17;13(12):e0202616. doi: 10.1371/journal.pone.0202616 (PMC6296539; doi:10.1371/journal.pone.0202616)
Supplement: S4 File — (DOCX) [file pone.0202616.s004.docx]

# Papers examining quality circles

Non-randomised controlled studies

**Dyrkorn, R., et al. (2016)**. "Peer academic detailing on use of antibiotics in acute respiratory tract infections. A controlled study in an urban Norwegian out-of-hours service." Scandinavian Journal of Primary Health Care 34(2): 180-185.

Using trained GPs to give peer academic detailing to colleagues in combination with open discussion on prescription, showed a significant change in prescription of antibiotics towards national guidelines. Key points Phenoxymethylpenicillin is the first choice for the most of respiratory tract infections when indicated. Despite the guidelines for the choice of antibiotics in Norway, general practitioners' choice often differs from these. We showed that a session of three times 45min of peer academic detailing changed significantly the choice of antibiotics towards the National Guidelines in an urban Norwegian out-of-hours service.

**Elward, K., et al. (2014)**. "Improving quality of care and guideline adherence for asthma through a group self-assessment module." Journal of the American Board of Family Medicine 27(3): 391-398.

Group Self-Assessment Module activities may be an effective method to increase physicians' knowledge of and adherence to clinical guidelines.

**Hartmann, P., et al. (1995)**. "Structured public health quality circle on the topic of diabetes management in general practice. [German] Strukturierte kassenarztliche Qualitatszirkel zum Thema Diabetikerbetreuung in der Praxis." Zeitschrift für ärztliche Fortbildung 89(4): 415-418.

The participation in a quality circle resulted in a significant and relevant improvement of the quality concerning the detection of diabetes related complications.

**Ioannidis, G., et al. (2007)** Canadian Quality Circle pilot project in osteoporosis: rationale, methods, and feasibility. Canadian family physician Médecin de famille canadien 1694-1700

Findings from the pilot study showed that the Canadian Quality Circle project was well designed and well received. Use of QCs appeared to be feasible for transferring knowledge and giving physicians an opportunity to analyze work-related problems and develop solutions to them.

**Ioannidis, G., et al. (2009).** "The utilization of appropriate osteoporosis medications improves following a multifaceted educational intervention: the Canadian quality circle project (CQC)." BMC Medical Education 9: 54.

QCs methodology was successful in increasing physicians' appropriate use of osteoporosis medications in accordance with Osteoporosis Canada guidelines.

**Mahlknecht, A., et al. (2016).** "Improving quality of care in general practices by self-audit, benchmarking and quality circles." Wien Klin Wochenschr 128(19-20): 706-718.

The intervention was able to improve measured quality of care. Barriers were detected that should be considered in a possible implementation of quality control programs.

**Niquille, A., et al. (2010)**. "The nine-year sustained cost-containment impact of swiss pilot physicians-pharmacists quality circles." Ann Pharmacother 44(4): 650-657.

The physicians-pharmacists quality circles work process has yielded sustainable results, such as significant cost savings, higher penetration of generics and reflection on patient safety, and the place of "new" drugs in therapy. The PPQCs may also constitute a solid basis for implementing more comprehensive collaborative programs, such as medication reviews, adherence-enhancing interventions, or disease management approaches.

**Richards, D., et al. (2003).** "Do clinical practice education groups result in sustained change in GP prescribing?" Family Practice 20(2): 199-206.

The results support a positive effect of the education strategy on prescribing behaviour in the intervention group for most outcomes measured. The effect seen is statistically significant, sustained and is in addition to any effect of the other pharmaceutical educational initiatives already undertaken by the independent GPs association.

**Riou, F., et al. (2007).** "Results of a 12-month quality-circle prescribing improvement programme for GPs." Br J Gen Pract 57(540): 574-576.

The reduction in drug expenditure exceeded the cost of the programme, although variations in size effects were observed among the settings.

**Wensing, M., et al. (2004).** "Quality circles to improve prescribing patterns in primary medical care: what is their actual impact?" J Eval Clin Pract 10(3): 457-466.

Large-scale application of quality circles had intended effects on prescribing decisions in primary care in Germany. The effects found in this study may reflect better what improvements can be achieved than randomized trials of similar interventions.

**Wensing, M., et al. (2009).** "Quality circles to improve prescribing of primary care physicians. Three comparative studies." Pharmacoepidemiol Drug Saf 18(9): 763-769.

Quality circles had a modest effect on prescribing quality and costs. If widely implemented, they could have nationwide impact on the quality and costs of prescribing in primary care.

Randomised controlled trials

**Gjelstad, S., et al. (2013)**. "Improving antibiotic prescribing in acute respiratory tract infections: cluster randomised trial from Norwegian general practice (prescription peer academic detailing (Rx-PAD) study)." BMJ 347: f4403.

The intervention led to improved antibiotic prescribing for respiratory tract infections in a representative sample of Norwegian general practitioners, and the courses were feasible to the general practitioners.

**Goldberg, H. I., et al. (1998).** "A randomized controlled trial of CQI teams and academic detailing: can they alter compliance with guidelines?" Joint Commission Journal on Quality Improvement 24(3): 130-142.

The academic detailing techniques and the continuous quality improvement teams evaluated were generally ineffective in improving guideline compliance and clinical outcomes regarding the primary care of hypertension and depression.

**Jager, C., et al. (2017)**. "Impact of a tailored program on the implementation of evidence-based recommendations for multimorbid patients with polypharmacy in primary care practices-results of a cluster-randomized controlled trial." Implementation Science 12(1): 8.

The tailored program may improve implementation of medication counselling and brown bag review whereas the use of medication lists and medication reviews did not improve. No effect of the tailored program on the combined primary outcome could be substantiated. Due to limitations of the study, results have to be interpreted carefully. The factors facilitating and hindering successful implementation will be examined in a comprehensive process evaluation.

**Lagerlov, P., et al. (2000)**. "Improving doctors' prescribing behaviour through reflection on guidelines and prescription feedback: a randomised controlled study." Qual Health Care 9(3): 159-165.

Deriving quality criteria of prescribing by discussing guideline recommendations gave the doctors a basis for judging their treatment of individual patients as acceptable or unacceptable. Presented with feedback on their own prescribing, they learned what they did right and wrong. This provided a foundation for improvement and the process thus instigated resulted in the doctors providing better quality patient care.

**Rognstad, S., et al. (2013).** "Prescription peer academic detailing to reduce inappropriate prescribing for older patients: a cluster randomised controlled trial." Br J Gen Pract 63(613): e554-562.

Educational outreach visits with feedback and audit, using GPs as academic detailers in GPs' CME groups, reduced PIPs for older patients aged >/=70 years in general practice.

**Schneider, A., et al. (2008).** "Impact of quality circles for improvement of asthma care: results of a randomized controlled trial." J Eval Clin Pract 14(2): 185-190.

Quality circles working with individualized feedback are effective at improving asthma care. The trial may have been underpowered to detect specific benchmarking effects. Further research is necessary to evaluate strategies for improving the self-management of asthma patients.

**Verbakel, N. J., et al. (2015)**. "Effects of patient safety culture interventions on incident reporting in general practice: A cluster randomised trial a cluster randomised trial." British Journal of General Practice 65(634): e319-e329.

Educating staff and facilitating discussion about patient safety culture in their own practice leads to increased reporting of incidents. It is beneficial to invest in a team-wise effort to improve patient safety.

**Verstappen, W. H., et al. (2003)**. "Effect of a practice-based strategy on test ordering performance of primary care physicians: a randomized trial." JAMA 289(18): 2407-2412.

In this study, a practice-based, multifaceted strategy using guidelines, feedback, and social interaction resulted in modest improvements in test ordering by primary care physicians.

**Verstappen, W. H., et al. (2004).** "Improving test ordering in primary care: the added value of a small-group quality improvement strategy compared with classic feedback only." Ann Fam Med 2(6): 569-575.

Compared with only disseminating comparative feedback reports to primary care physicians, the new strategy of involving peer interaction and social influence improved the physicians' test-ordering behavior. To be effective, feedback needs to be integrated in an interactive, educational environment.

**Verstappen, W. H., et al. (2004).** "Comparing cost effects of two quality strategies to improve test ordering in primary care: a randomized trial." Int J Qual Health Care 16(5): 391-398.

On the basis of our findings, including the expected non-monetary benefits, we recommend further long-term effect and cost-effect studies on the implementation of the quality strategy.

**Vervloet, M., et al. (2016).** "Reducing antibiotic prescriptions for respiratory tract infections in family practice: results of a cluster randomized controlled trial evaluating a multifaceted peer-group-based intervention." NPJ Primary Care Respiratory Medicine 26: 15083.

This multifaceted peer-group-based intervention was effective in reducing the number of RTI-related antibiotic prescriptions for adolescents and adults. To affect antibiotic prescribing in children other methods are needed.

**Welschen, I., et al. (2004).** "Effectiveness of a multiple intervention to reduce antibiotic prescribing for respiratory tract symptoms in primary care: randomised controlled trial." BMJ 329(7463): 431.

A multiple intervention reduced prescribing rates of antibiotics for respiratory tract symptoms while maintaining a high degree of satisfaction among patients. Further research should focus on the sustainability and cost effectiveness of this intervention.

**Wilcock, J., et al. (2013).** "Tailored educational intervention for primary care to improve the management of dementia: The EVIDEM-ED cluster randomized controlled trial." Trials 14 (1) (no pagination)(397).

The trial was timely, coinciding with financial incentives for dementia management in general practice (through the Quality Outcomes Framework); legal imperatives (in the form of the Mental Capacity Act 2005); policy pressure (The National Dementia Strategy 2009); and new resources (such as dementia advisors) that increased the salience of dementia for general practitioners. Despite this the intervention did not alter the documentation of clinical management of patients with dementia in volunteer practices, nor did it increase case identification.

Mixed methods study

**Ter Brugge, B. P. H., et al. (2017)**. "The use of evidence during group meetings of Dutch general practitioners." Education for Primary Care 28(6): 307-312.

In the Netherlands, quality circles seem to be more goal-oriented than learning oriented. Learning discussions about controversies in clinical research or about the integration of evidence, patient values and clinical expertise occurred infrequently. To harvest the potential value of group meetings for EBM learning, quality circles in their present design are not optimal.

Qualitative studies

**Andres, E., et al. (2015)**. "20 years of quality circles for family practitioners - Stocktaking and perspectives: A workshop report. [German]." Zeitschrift fur Allgemeinmedizin 91(2): 66-70.

Measures were identified to support and improve QC-work: Among others, structured and evidence-based materials on different QC-topics should be available on demand for QC moderators. Furthermore, a good self-confidence of FPs was identified as fundamental for a fruitful exchange on equal footing between FPs and medical specialists. To further improve and refine QCs a lot of issues still remain to be addressed by all stakeholders involved.

**Fisher, D. M., et al. (2013)**. "Engagement of groups in family medicine board maintenance of certification." Journal of the American Board of Family Medicine: JABFM 26(2): 149-158.

Practice-level collaboration, access to a practice coach, flexibility in choosing and focusing improvement projects, tailored support, and involvement with professional affiliations can enhance the Part IV MOC process. Specialty boards are likely to discover productive opportunities from working with practices, professional organizations, and health care systems to support intra- and interpractice collaborative QI work that uses Part IV Maintenance of Certificatio requirements to motivate practice improvement.

**Francois, P., et al. (2013).** "[Peer groups: a model for the continuous professional development in general practice]." Presse Medicale 42(1): e21-27.

This study showed that peer group activity was growing in Isere. It was widely welcomed by GPs, who wondered in which way those groups could satisfy the obligations of professional development mentioned in the 2009 French law: Hospital, Patient, Health, Territory (HPST).

**Frich, J., et al. (2010)**. "General practitioners and tutors' experiences with peer group academic detailing: a qualitative study." BMC Family Practice 11(1): 12.

GPs and tutors experienced peer group academic detailing as a suitable method to discuss and learn more about pharmacotherapy. An important outcome for GPs was being more reflective about their prescriptions. Disclosure of inappropriate prescribing can cause distress in some doctors, and tutors must be prepared to recognise and manage such reactions.

**Gehring, K., et al. (2013).** "Safety climate and its association with office type and team involvement in primary care." International Journal for Quality in Health Care 25(4): 394-402.

Results indicate that frequent quality circle participation and team meetings involving all team members are effective ways to strengthen safety climate in terms of team-based strategies and activities in error prevention.

**Gehring, S. C., et al. (2017)**. "Structured pharmacotherapy in multimorbid seniors - A pilot project. [German]." Zeitschrift fur Allgemeinmedizin 93(6): 266-270.

The algorithm was feasible to use. To what extent the observed reduction of the number of prescriptions is causally related to the algorithm should be tested within a controlled design.

**Jenson, C. M., et al. (2006)**. "Is small-group education the key to retention of sessional GPs?" Education for Primary Care 17(3): 218-226.

The association between educational support and retention should be studied for other members of the primary care team, health professionals outside general practice and healthcare systems outside the NHS and the UK.

**Nielsen, H. G. and A. S. Davidsen (2017).** "Witnesses in the consultation room - Experiences of peer group supervision." Education for Primary Care 28(5): 258-264.

Participation seemed to improve communication skills and the ability to take a more patient-centred approach. It increased job satisfaction and prevented burnout. A non-judgemental environment, respect and acknowledgement of all participants were important for developing confidence and for supporting personal and professional development.

**Overton, G. K., et al. (2009).** "The Practice-based Small Group Learning programme: experiences of learners in multi-professional groups." J Interprof Care 23(3): 262-272.

The learning process in the groups came close to transformative learning--there were changes in perspectives, acquisition of new knowledge and increased self-esteem. The appropriateness of the PBSGL approach for the CPD of mixed groups of GPs and PNs is discussed.

**Roberts, C. M., et al. (2012).** "A randomized trial of peer review: the UK National Chronic Obstructive Pulmonary Disease Resources and Outcomes Project: three-year evaluation." J Eval Clin Pract 18(3): 599-605.

The findings demonstrate significant change in service provision over 3 years in both control and intervention sites with great variability in both groups. The combined quantitative and qualitative findings indicate that targeted mutual peer review is associated with improved quality of care, improvements in service delivery and with changes within departments that promote and are precursors to quality improvement.

**Sommers, L. S., et al. (2007).** "Practice inquiry: clinical uncertainty as a focus for small-group learning and practice improvement." J Gen Intern Med 22(2): 246-252.

Ongoing clinician involvement suggests that Practice Inquiry is a feasible, acceptable, and potentially useful set of PBLI methods. Two of the Practice Inquiry's group learning tasks received comparatively less focus: integrating research evidence with clinical experience and tracking dilemma case outcomes. Future work should focus on reducing the methodological limitations of a demonstration effort and examining factors affecting clinician participation. Set-aside work time for clinicians, or other equally potent incentives, will be necessary for the further elaboration of these PBLI methods aimed at managing uncertainty.

**Watkins, C., et al. (2004)**. "Factors affecting feasibility and acceptability of a practice-based educational intervention to support evidence-based prescribing: a qualitative study." Fam Pract 21(6): 661-669.

The study indicates the importance of preparing the practice adequately, including providing protected time for all GPs to attend the educational intervention. Scenarios and the structure of the sessions need to make more explicit the links between everyday practice and published evidence of effectiveness. Emphasis on cost-effectiveness may be counterproductive and wider benefits need to be emphasized. We have also identified the skill profile of the facilitator role. Our study indicates a need for a clearer understanding of GPs' perception of clinical autonomy and how this conflicts with the goal of agreement on practice guidelines for treatment. The intervention is now ripe for further development, perhaps by integrating it with other interventions to change professional behaviour. The improved intervention should then be evaluated in a randomized controlled trial.

Systematic reviews on elements of quality circles

**Arnold, S. and S. Straus (2005)** Interventions to improve antibiotic prescribing practices in ambulatory care. Cochrane Database of Systematic Reviews DOI: 10.1002/14651858.CD003539.pub2

Multi‐faceted interventions where educational interventions occur on many levels may be successfully applied to communities after addressing local barriers to change. These were the only interventions with effect sizes of sufficient magnitude to potentially reduce the incidence of antibiotic‐resistant bacteria.

**Baker, R., et al. (2010)** Tailored interventions to overcome identified barriers to change: effects on professional practice and health care outcomes. Cochrane Database of Systematic Reviews DOI: 10.1002/14651858.CD005470.pub2

The findings indicate that tailored interventions can change professional practice. As yet, there is insufficient evidence on the most effective approaches to tailoring, including how barriers should be identified and how interventions should be selected to address the barriers.

**Baker, R., et al. (2015)**. "Tailored interventions to address determinants of practice." Cochrane Database Syst Rev 4: Cd005470.

Tailored implementation can be effective, but the effect is variable and tends to be small to moderate.

**Baskerville, N. B., et al. (2012).** "Systematic Review and Meta-Analysis of Practice Facilitation Within Primary Care Settings." The Annals of Family Medicine 10(1): 63-74.

Practice facilitation has a moderately robust effect on evidence-based guideline adoption within primary care. Implementation fidelity factors, such as tailoring, the number of practices per facilitator, and the intensity of the intervention, have important resource implications.

**Bowie, P., et al. (2008)**. "A review of the current evidence base for significant event analysis." Journal of Evaluation in Clinical Practice 14(4): 520-536.

A chasm exists between the high expectations for SEA and the lack of evidence of its impact. Significant event analysis may have some merit as a team-based educational tool.

**Cadogan, S. L., et al. (2015)**. "The effectiveness of interventions to improve laboratory requesting patterns among primary care physicians: a systematic review." Implement Sci 10: 167.

Interventions such as educational strategies, feedback and changing test order forms may improve the efficient use of laboratory tests in primary care; however, the level of evidence is quite low and the quality is poor. The reproducibility of findings from different laboratories is also difficult to ascertain from the literature.

**Davis, D., et al. (1999)**. "Impact of formal continuing medical education: Do conferences, workshops, rounds, and other traditional continuing education activities change physician behavior or health care outcomes?" JAMA 282(9): 867-874.

Our data show some evidence that interactive CME sessions that enhance participant activity and provide the opportunity to practice skills can effect change in professional practice and, on occasion, health care outcomes. Based on a small number of well-conducted trials, didactic sessions do not appear to be effective in changing physician performance.

**Davis, D. A., et al. (2006)**. "Accuracy of physician self-assessment compared with observed measures of competence: a systematic review." JAMA 296(9): 1094-1102.

While suboptimal in quality, the preponderance of evidence suggests that physicians have a limited ability to accurately self-assess. The processes currently used to undertake professional development and evaluate competence may need to focus more on external assessment.

**Dogherty, E. J., et al. (2010).** "Facilitation as a Role and Process in Achieving Evidence-Based Practice in Nursing: A Focused Review of Concept and Meaning." Worldviews on Evidence-Based Nursing 7(2): 76-89.

Further understanding of what facilitators are actually doing to enable changes in practice based on research findings will provide the groundwork for the design and evaluation of practical strategies for evidence-based practice. Research is needed to clarify how facilitation may be used to implement change in practice along with evaluation of the effectiveness of various approaches.

**Farmer A., P., et al. (2008)** Printed educational materials: effects on professional practice and health care outcomes. Cochrane Database of Systematic Reviews DOI: 10.1002/14651858.CD004398.pub2

The results of this review suggest that when compared to no intervention, PEMs when used alone may have a beneficial effect on process outcomes but not on patient outcomes. Despite this wide of range of effects reported for printed educational materials, clinical significance of the observed effect sizes is not known.

**Flodgren, G., et al. (2011)**. "Local opinion leaders: effects on professional practice and health care outcomes." Cochrane Database Syst Rev(8): Cd000125.

Opinion leaders alone or in combination with other interventions may successfully promote evidence-based practice, but effectiveness varies both within and between studies. These results are based on heterogeneous studies differing in terms of type of intervention, setting, and outcomes measured. In most of the studies the role of the opinion leader was not clearly described, and it is therefore not possible to say what the best way is to optimise the effectiveness of opinion leaders.

**Forsetlund, L., et al. (2009)**. "Continuing education meetings and workshops: effects on professional practice and health care outcomes." Cochrane Database Syst Rev(2): Cd003030.

Educational meetings alone or combined with other interventions, can improve professional practice and healthcare outcomes for the patients. The effect is most likely to be small and similar to other types of continuing medical education, such as audit and feedback, and educational outreach visits. Strategies to increase attendance at educational meetings, using mixed interactive and didactic formats, and focusing on outcomes that are likely to be perceived as serious may increase the effectiveness of educational meetings. Educational meetings alone are not likely to be effective for changing complex behaviours.

**Giguere, A., et al. (2012).** "Printed educational materials: effects on professional practice and healthcare outcomes." Cochrane Database Syst Rev 10: CD004398.

The results of this review suggest that when used alone and compared to no intervention, PEMs may have a small beneficial effect on professional practice outcomes. There is insufficient information to reliably estimate the effect of printed educational materials on patient outcomes, and clinical significance of the observed effect sizes is not known. The effectiveness of printed educational materials compared to other interventions, or of printed educational materials as part of a multifaceted intervention, is uncertain.

**Gill, P. S., et al. (1999).** "Changing doctor prescribing behaviour." Pharm World Sci 21(4): 158-167.

We identified 79 eligible studies which described 96 separate interventions to change prescribing behaviour. Of these interventions, 49 (51%, 41%-61%) showed a positive significant change compared to the control group but interpretation of specific interventions is limited due to wide and overlapping confidence intervals.

**Grimshaw, J. M., et al. (2012).** "Disseminating and implementing guidelines: article 13 in Integrating and coordinating efforts in COPD guideline development. An official ATS/ERS workshop report." Proc Am Thorac Soc 9(5): 298-303.

The Knowledge to Action cycle proposed by Graham and colleagues provides a useful framework for planning dissemination and implementation activities that emphasize the need for tailored approaches based on an assessment of local barriers. There are a broad range of interventions that are generally effective at improving the uptake of evidence. The best intervention depends on likely barriers, available resources, and other practical considerations. Financial interventions (such as pay for performance) appear to be as effective as other interventions that aim to change professional behaviour.

**Harris J., K. K., et al. (2011)**. "Are journal clubs effective in supporting evidence-based decision making? A systematic review. BEME Guide No. 16." Medical Teacher 33(1): 9-23.

The effectiveness of JCs in supporting evidence-based decision making is not clear.

**Ivers, N., et al. (2012)** Audit and feedback: effects on professional practice and healthcare outcomes. Cochrane Database of Systematic Reviews DOI: 10.1002/14651858.CD000259.pub3

Audit and feedback generally leads to small but potentially important improvements in professional practice. The effect of audit and feedback on professional behaviour and on patient outcomes ranges from little or no effect to a substantial effect. Audit and feedback may be most effective when: 1. the health professionals are not performing well to start out with; 2. the person responsible for the audit and feedback is a supervisor or colleague; 3. it is provided more than once; 4. it is given both verbally and in writing;5. it includes clear targets and an action plan. In addition, the effect of audit and feedback may be influenced by the type of behaviour it is targeting.

**O'Brien, M., et al. (2007)** Educational outreach visits: effects on professional practice and health care outcomes. Cochrane Database of Systematic Reviews DOI: 10.1002/14651858.CD000409.pub2

Educational outreach visits alone or when combined with other interventions have effects on prescribing that are relatively consistent and small, but potentially important. Their effects on other types of professional performance vary from small to modest improvements, and it is not possible from this review to explain that variation.

**O'Brien, M. A., et al. (2001)**. "Continuing education meetings and workshops: effects on professional practice and health care outcomes." Cochrane Database Syst Rev(2): CD003030.

Interactive workshops can result in moderately large changes in professional practice. Didactic sessions alone are unlikely to change professional practice.

**Parmelli, E., et al. (2011)** The effectiveness of strategies to change organisational culture to improve healthcare performance. Cochrane Database of Systematic Reviews DOI: 10.1002/14651858.CD008315.pub2

It is not possible to draw any conclusions about the effectiveness of strategies to change organisational culture because no studies were found that fulfilled the methodological criteria for this review.

**Zaher, E. and S. Ratnapalan (2012).** "Practice-based small group learning programs: systematic review." Can Fam Physician 58(6): 637-642, e310-636.

Current evidence suggests that Problem Based Small Group Learning is a promising method of continuing professional development for GPs. Such programs can be adapted according to learning needs. Future studies that focus on the changes in practice effected by Problem Based Small Group Learning will strengthen the evidence for this form of learning and motivate physicians and institutions to adopt it.
